# Learned Insignificance of Credibility Signs

**DOI:** 10.1111/cogs.70102

**Published:** 2025-08-14

**Authors:** Viktoria Kainz, Justin Sulik, Sonja Utz, Torsten Enßlin

**Affiliations:** ^1^ Max Planck Institute for Astrophysics Garching; ^2^ Faculty of Physics Ludwig Maximilian University of Munich; ^3^ Cognition, Values and Behavior Ludwig Maximilian University of Munich; ^4^ Leibniz‐Institut für Wissensmedien Tübingen; ^5^ Department of Psychology, Faculty of Science University of Tübingen

**Keywords:** Social information, Cognition, Credibility, Misinformation, Emergent cognitive biases, Bayesian reasoning, Agent‐based model

## Abstract

A large part of how people learn about their shared world is via social information. However, in complex modern information ecosystems, it can be challenging to identify deception or filter out misinformation. This challenge is exacerbated by the existence of a dual‐learning problem whereby: (1) people draw inferences about the world, given new social information; and simultaneously (2), they draw inferences about how credible various sources of information are, given social cues and previous knowledge. In this context, we investigate how social influence and individual cognitive processing interact to explain how one might lose the ability to reliably assess information. Crucially, we show how this happens even when individuals engage in rational belief updating and have access to objective cues of deception.

Using an agent‐based model, the Reputation Game Simulation, we show that mere misinformation is not the problem: The dual‐learning problem can be solved successfully with limited Bayesian reasoning, even in the presence of deceit. However, when certain agents consistently engage in fully deceptive behavior, intentionally distorting information to serve nonepistemic goals, this can lead nearby agents to unlearn or discount objective cues of credibility. This is an emergent delusion‐like state, wherein false beliefs resist correction by true incoming information. Further, we show how such delusion‐like states can be rehabilitated when agents who had previously lost the ability to discern cues of credibility are put into new, healthy—though not necessarily honest—environments.

Altogether, this suggests that correcting misinformation is not the optimal solution to epistemically toxic environments. Though difficult, socially induced cognitive biases can be repaired in healthy environments, ones where cues of credibility can be relearned in the absence of nonepistemic communication motives.

## 1 Introduction

To learn about the world, we humans depend heavily on social information—information gained from others, which we often accept on the basis of testimony—rather than evidence which we gain through our own direct experience. It is challenging to build an accurate picture of the world in this way because we inhabit a complex informational ecology: Individuals encounter numerous sources of information, ranging from intimate social networks to vast media platforms or from peers to experts.

These sources vary widely in their epistemic trustworthiness (Hendriks, Distel, Engelke, Westmattelmann, & Wintterlin, 2021; O'Neill, 2018). For instance, with the rise of the internet and especially social media, people are exposed to immense quantities of information, including misinformation (Vosoughi, Roy, & Aral, 2018; Wang, McKee, Torbica, & Stuckler, 2019).^1^ Belief in misinformation has partly been explained by aspects of human psychology, such as motivated reasoning, unreflective processing, or inattention (Acerbi, 2019; Bronstein, Pennycook, Bear, Rand, & Cannon, 2019; Pennycook et al., 2021; Scherer et al., 2021; Tandoc Jr, 2019). However, individual psychology is only a partial explanation because it initially seems hard to reconcile with the contradictory claims of widespread epistemic vigilance (Mercier, 2017).

A fuller picture requires that we situate individual cognitive processes in dynamic social contexts (Dingemanse et al., 2023). In particular, anyone integrating social information into their understanding of the world faces a dual learning problem: They must learn about the world using information from social sources and must simultaneously learn how reliable each source is (Barrett, Skyrms, & Mohseni, 2019), all while information spreads across—and is influenced by—social networks. Information sources are often not independent, showing multiple paths of mutual influence and making it especially challenging to evaluate the evidentiary value of information in interactive social contexts (Hahn, Harris, & Corner, 2016; Lazer et al., 2018; Tokita, Guess, & Tarnita, 2021; Yousif, Aboody, & Keil, 2019).

The consequences of doing this poorly can be catastrophic at both individual and group levels, as when misinformation and mistrust lead to individual rejection of scientific consensus about climate change and vaccine safety, or to ill‐advised social policies and inadequate societal responses to global challenges.

In this context, one puzzle is to understand how individuals form beliefs that persistently conflict with evidence that is both readily available and objectively useful. For instance, even though the evidence for climate change is ample, with multiple kinds of evidence aligning to support consensus climate models, many individuals reject that evidence outright. The same individuals are also likely to distrust scientific sources (Azevedo & Jost, 2021; Lewandowsky & Oberauer, 2021; Sinatra & Hofer, 2016). The problem is that, if an individual's beliefs about a source's trustworthiness become divorced from objective cues of the credibility of that source's claims (Hahn et al., 2016; Levy, 2019), then that individual could get stuck in a belief state thoroughly at odds with reality.

We describe this as a delusion‐like state because—although we are explicitly not addressing a clinical phenomenon, but rather a phenomenon in the general population—delusions are “fixed beliefs that are not amenable to change in light of conflicting evidence” (American Psychological Association, 2022). Even in nonclinical populations, proneness to delusion‐like beliefs is associated with increased belief in fake news (Bronstein, Pennycook, Bear, Cannon, & Rand, 2018), and cognitive biases implicated in delusion‐like beliefs are associated with rejection of evidence‐based medicine (Bryden, Browne, Rockloff, & Unsworth, 2018; Sunyik & Čavojová, 2023).

We aim to understand the causes of delusion‐like states in systems of interacting simulated agents, and in particular, cases where individuals learn to discount otherwise valuable evidence. We further aim to identify potential rehabilitations once an agent is in such a state, pointing to potential solutions to the problem.

### 1.1 Social versus cognitive explanations

Whereas previous explanations of similar phenomena are typically grounded either in social contexts or in individual cognition, we are searching for explanations in the interaction between social and cognitive domains. This approach is motivated by the likelihood of feedback loops between the social environment and individuals' information processing strategies, and it builds on a tradition of using physics models of complex dynamic systems to understand social relationships (“sociophysics”), here by investigating emergent cognitive biases from interactions with other agents.

Given the flexible and error‐prone nature of social learning (Boyd & Richerson, 1985; Barrett et al., 2019; Derex, Perreault, & Boyd, 2018; Rendell et al., 2010) and given path dependence in evolving cultural systems (Bikhchandani, Hirshleifer, & Welch, 1992; Salganik, Dodds, & Watts, 2006), some explanations of socially learned false beliefs focus on the social contexts themselves. Efficient spread of social information can cause even rational agents to converge prematurely on local optima (Zollman, 2007, 2010) or boosting error rates (Hahn, von Sydow, & Merdes, 2019). Over time, social influence can lead agents to form echo chambers where different clusters of rational agents nonetheless hold incompatible beliefs (Madsen, Bailey, & Pilditch, 2018). Further, the epistemic performance of a particular (boundedly) rational strategy is contingent on the distribution of strategies across the group (Mayo‐Wilson, Zollman, & Danks, 2013), including on emerging network structures (Barrett et al., 2019). Finally, social communication channels typically involve message compression (e.g., when claims are transmitted without supporting reasons (Rahwan, Krasnoshtan, Shariff, & Bonnefon, 2014)), so truthful claims may fail to spread throughout a network.

A second thread of research focuses on cognitive traits of individuals. Susceptibility to antiempirical beliefs is associated with individual differences in preferences for certain kinds of explanations (Boudry, Blancke, & Pigliucci, 2015; Lindeman, Svedholm‐Häkkinen, & Riekki, 2022; Prike, Arnold, & Williamson, 2020; Sulik, Ross, & McKay, 2020), reduced or biased data gathering strategies (Bronstein, Kummerfeld, MacDonald III, & Vinogradov, 2022; Rodríguez‐Ferreiro & Barberia, 2021; Sanchez & Dunning, 2020; Sulik & McKay, 2021), or in tendencies to engage in low‐effort processing (Gervais, 2015; Pennycook & Rand, 2021).

However, individual cognitive traits need not be irrational for a group to form conflicting beliefs. For instance, rational agents can disagree strongly about empirical matters if they start with different priors or different models of the data‐generating process (Cook & Lewandowsky, 2016; Jern, Chang, & Kemp, 2014; Navarro, Perfors, Kary, Brown, & Donkin, 2018). Such approaches thus include a causal pathway from individual cognitive traits to emerging group phenomena, meaning that group‐level outcomes can be explained by individuals' behavior.

Our approach here also considers the reverse: a causal pathway from social influence to emergent cognitive states. Work on collective decision‐ making has shown that social information, such as information about the decision‐making strategies or status of others, can reinforce or amplify individual cognitive biases (Bahrami et al., 2010; Hardy, Thompson, Krafft, & Griffiths, 2022; Sulik, Efferson, & McKay, 2021), leading to suboptimal individual and group decisions. In a similar vein, we allow in our model for a feedback loop between social information and individual cognition, such that we expect to see self‐amplifying biases contributing to delusion‐like states.

To study this interplay between the individual and group level, we will look at small groups of just 3 agents. This allows us to observe the effects of both individual cognitive traits (as they are still strong and important enough) and the mutual influence of the agents on each other (which is already observable in a 3‐agent system), while we focus on how individuals learn (or fail to learn) about social signals of credibility. Effects of group opinion formation that come on top in larger networks are out of scope of this work and will be part of future research.

### 1.2 Learning to discount cues of credibility

Our approach here builds on a sociophysics model, the Reputation Game Simulation (Enßlin, Kainz, & Bœhm, 2022), wherein agents send and receive messages. The messages consist of information about each others' honesty (their “reputation”). Thus, agent a might communicate to agent b about how honest agent c is, in which case, a is b’s source of some social information. Crucially, the messages may be accompanied by an objective cue of the source's honesty. This evidential cue, which we label a “blush” for ease of reference, sometimes appears when an agent lies but never when it tells the truth. The frequency of this cue is a fixed parameter of the simulation.

In the original Reputation Game, all agents knew the frequency with which any lie would be accompanied by a blush. In the current study, the agents have to learn that frequency. Thus, agents now face the aforementioned dual‐learning problem of trying to understand the world (here: how honest all agents are) while simultaneously learning cues to the reliability of social information about that world (how often lies are cued by blushes). This speaks to the problem we identified above because an agent may learn to discount the evidential value of the blush cue when the source is observed to be blushing if the learner has a strong‐enough prior that the source is honest.

We previously described the situation wherein an agent's beliefs about a source's honesty become divorced from cues to the evidential value of that source's information as a delusion‐like state. In particular, this has parallels to the Bias Against Disconfirmatory Evidence (BADE), a bias implicated in the maintenance of delusions and delusion‐like beliefs (Bronstein, Pennycook, Joormann, Corlett, & Cannon, 2019; McLean, Mattiske, & Balzan, 2017; Menon et al., 2013; Woodward, Moritz, Cuttler, & Whitman, 2006), wherein people are prone to disregard new evidence that conflicts with their previously held (false) beliefs.

However, whereas the previous literature treats BADE as a (presumably stable) trait of individuals and focuses on mismatches between new information and prior beliefs observed in clinical contexts (common among people diagnosed with schizophrenia), our model examines a learned bias and incorporates an explicit cue of evidential value (the blush signal) that accompanies new information. For clarity, we thus refer to “Learned Insignificance of Credibility Signs” (LICS) rather than BADE.

The LICS is thus a suitable candidate phenomenon given our aim to explain emergent delusion‐like states, whereby individuals could get stuck—whether temporarily or permanently—in belief states at odds with reality as a result of a dynamic interaction between individual cognition and social contexts. Further, this counts as a causal pathway from social context to individual cognition because such an agent would have learned to discount a cue that has objective evidential value due—in part, at least—to social information.

### 1.3 Rehabilitating biased individuals

Rehabilitating those with antiempirical beliefs can be challenging. For instance, if someone has a high prior belief that scientists are lying, then informing them that there is a high degree of consensus about climate change among scientists may provoke a backlash, actually decreasing belief in climate change rather than the intended outcome of increasing it (Cook & Lewandowsky, 2016).

This is one reason we investigate the dynamic interaction between individual cognition and social context, because rehabilitation may have as much to do with changing the social context as it does with changing someone's beliefs. Sociological evidence shows that placing someone who is sceptical of climate change in a new social context (e.g., when they leave the family environment and go away to college) may help them learn to recalibrate their cues of trustworthiness and thereby come to believe in climate change (Haltinner, Sarathchandra, Ziegler, & Stuart, 2022). Thus, we examine two types of simulation runs:

1. **Learning runs**: Agents with different parameter settings (see formal specifications below) send and receive messages sometimes accompanied by blush cues as they simultaneously try to learn about each others' reputations for honesty and about the evidential value of the blush clues; and
2. **Rehabilitation runs**: Agents who lost the ability to discern the evidential value of blushes during a learning run are placed in a healthier social environment (defined below) to see when and how they recover the ability to discern others' honesty in light of the blush cues.

## 2 Model

The Reputation Game Simulation (RGS) is an agent‐based communication model. In each simulation run, a group of agents (here: 3 agents) repeatedly communicate with each other about their mutual reputations for honesty. Each agent thereby has its own, intrinsic honesty, which is the probability of that agent telling the truth in any given conversation. The estimation of one agent a about the intrinsic honesty of another agent b is agent b’s reputation in the eyes of a. At the same time, this reputation is the agents' only conversation topic: They talk (honestly or not) about how honest they believe someone else or themselves to be.^2^

In doing so, the main goal of the agents is to appear as honest as possible in the others' eyes. While one way of achieving this is to actually be honest, a second way—often associated with more power—is deception. As a consequence, the agents simultaneously face a second goal in order to handle the potentially false information they receive: They need to estimate the others' true, intrinsic honesties accurately to judge the credibilities of their respective statements in the first place.

For both goals, it is crucial that agents learn as much as possible from all the data that is available to them. This data mostly consists of the reputational messages their conversation partners send to them. An additional kind of data is the evidential cue: Whenever an agent lies, there is a 10% risk that the lie will be accidentally revealed by “blushing.” This is analogous to any situational or nonverbal sign that humans might give when lying (including differences in body language, facial expression, tonality, wording, style, etc.), but ultimately, it is just a second information signal sent (if at all) in parallel with the reputational message. An agent will never blush while telling the truth, however. This way, a blushing cue (in theory) should be a clear and unambiguous sign of a lie and should help the agents orient themselves. Nevertheless, it is crucial for the agents to understand and interpret those conversations and additional hints correctly in order to master both simultaneous goals.

### 2.1 Belief representation

In order to understand how the agents exchange information, we have to introduce their internal belief representation. Most of the dynamics the agents have to deal with in the RGS are random events, each with two possible outcomes at a time: someone lies or says the truth; someone blushes or does not. Note, however, the intrinsic honesty of an agent is indeed a number out of the continuous interval [0,1]. Similarly, also the communicated messages about someone's honesty are continuous, as will be explained later. Moreover, the way a receiving agent interprets a message is usually anywhere between surely honest and surely dishonest, but still, there are only two possibilities (a message being honest or not) to which the agent has to assign probabilities for an individual event. Similarly, also for blushing or no‐blushing, there are only these two possibilities at a time, irregardless of how the receiver interprets them. When reasoning about the underlying frequency f of such two‐outcome‐events, the beta distribution is a natural way to describe agents' evolving knowledge about f with which one or the other event occurs. Thus, we model the agents' beliefs and uncertainties about the value of a frequency f as:

(1) $$\begin{array}{c} P\left(f|I\right)=\mathrm{Beta}\left(f|\mu +1,\lambda +1\right)=\frac{f^{\mu }{\left(1-f\right)}^{\lambda }}{B(\mu +1,\lambda +1)}, \end{array}$$

where B is the beta function and I=(μ,λ) is the knowledge that an agent has about the frequency f. It is determined by the two parameters μ and λ, which can be understood as the number of times a specific event occurred or did not occur, respectively. For example, when considering agent a’s representation of agent b’s honesty, μ and λ can be seen as the number of honest and dishonest statements that agent a has observed (or rather, inferred) from agent b in the past. From this distribution, one can also calculate the mean and the variance of a belief state, that is, the agents' current best guess $\bar{f}$ and their uncertainty σ around it. These are given by:

(2) $$\begin{array}{cc} \bar{f} & =\frac{\mu +1}{\mu +\lambda +2} \end{array}$$

(3) $$\begin{array}{cc} \sigma & =\sqrt{\frac{\left(\mu +1)(\lambda +1\right)}{\left(\mu +\lambda +3\right){\left(\mu +\lambda +2\right)}^{2}}}. \end{array}$$

**Fig. 1:**
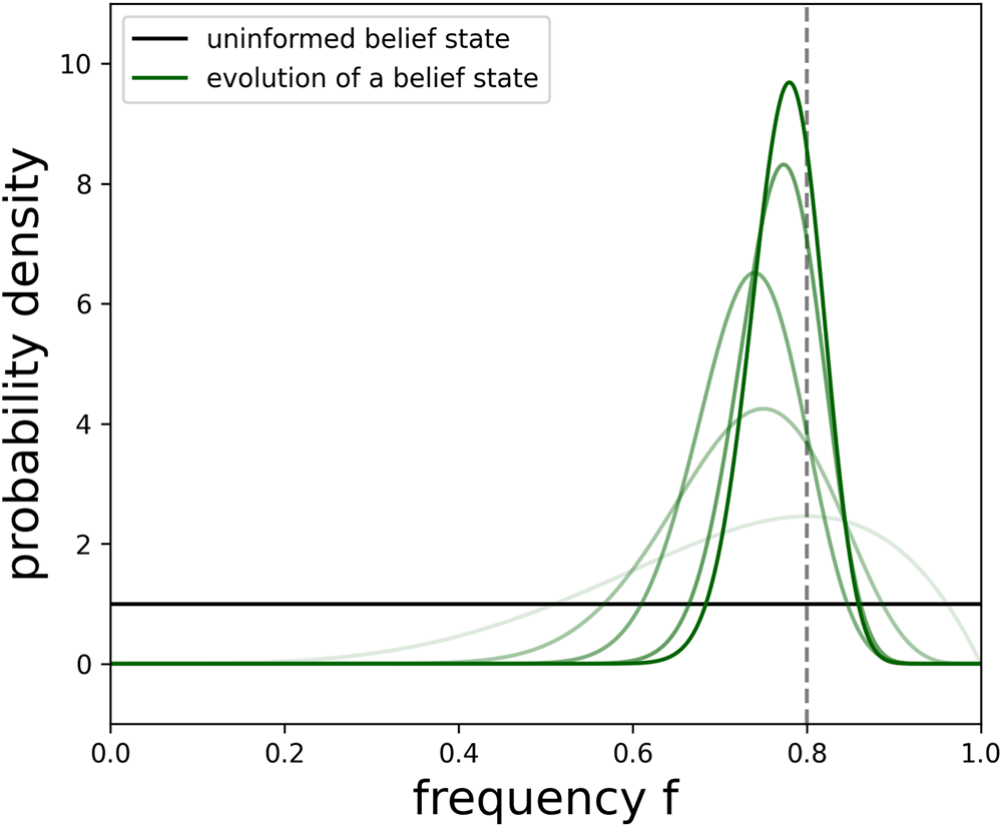
Examplary evolution of a belief state about a frequency f modeled as a beta distribution. The true frequency of an event happening in the underlying random experiment is f=0.8 (dashed line). By drawing more samples, that is, performing the random experiment more often, one gathers more and more data and thus better estimations of the true frequency. The black line shows an uninformed belief state where no sample has been drawn yet. The green curves show an exemplary evolution of a belief state that becomes more and more informed with the number of drawn samples (here 5, 20, 50, 75, and 100), indicated by an increasing intensity of the line.

Fig. 1 illustrates the evolution of such a learning process with increasing amounts of information.

### 2.2 Conversations

Conversations in the RGS follow a simple schema. First, some agent (the conversation initiator) randomly chooses a conversation partner (one of the other agents) and a conversation topic (the honesty of any agent, including itself or its conversation partner). Then, both conversation partners express their opinions on the chosen topic including their uncertainty. Each agent communicates honestly or lies based on its individual intrinsic honesty frequency. Thus, an agent with intrinsic honesty of 0.9 will have a 90% probability of telling the truth in a given conversation.

In case of an honest statement, the speaker simply transmits its current belief state in the form of the tuple (μ,λ) that specifies the full beta distribution that assigns every possible honesty value a probability. In case of a dishonest statement, they still communicate a full distribution given by the tuple (μ,λ), which is, however, no longer their true belief but can be any other values for both parameters. Thus, there are multiple options how to choose them. Following the main goal of appearing as honest as possible, as introduced above, we can deduce two subgoals that guide the agents when designing their lies: they want to appear convincing and to influence others in a way that increases their reputation. In order to meet both criteria, a lying agent starts constructing the message around its estimation of the receiver's current belief on the topic using their Theory of Mind^3^ (in order to sound convincing) and then shifts that belief, that is, the mean of the distribution, either to the positive (increasing μ by a certain value α) or to the negative (increasing λ by a certain value α)^4^ (in order to take influence). To determine the direction of this shift (positive or negative), we introduced an additional variable, friendship. Agents can either be friends or enemies (or neutral at the beginning of the simulation) and the lie direction depends on the current friendship status between the speaking agent and the topic, which may change during the simulation. Friends and enemies in the RGS are defined as agents who used to speak more positively/negatively about oneself than the average in past conversations. Thus, lies about friends (and themselves) are biased positively in order to increase their reputation in the eyes of others, while lies about enemies are biased negatively to decrease their reputation. Note: The friendship status between agents only influences the message construction but not the message comprehension. For the latter, the agents would need to keep track not only of their own, but also of others' friendships, which is currently not implemented.

Example: What happens if two agents Alice and Carol talk about a third one, Bob. Let us assume, Alice currently thinks that Bob has made 17 honest statements and 4 dishonest ones, for example, is rather honest. At the same time, Carol might have a worse picture of Bob, namely, 20 dishonest statements and only 5 honest ones. Now, Alice talks to Carol about Bob. In case Alice communicates honestly, the message would simply be (μ=17,λ=4), her true opinion. In case she lies, we have to additionally consider her friendship status with Bob. Let us assume she considers him a friend and wants to make a favorable statement. Since Carol's current belief about Bob is very bad, and assuming that Alice is roughly aware of this—say Alice believes Carol's opinion about Bob is (μ=5,λ=18) —Alice has to adapt the message such that it appears reasonable for Carol. Thus, the communicated message would be (μ=5+2,λ=18), that is, Alice's current best guess on Carol's opinion, modified by α=2 positive statements in order to increase Bob's reputation. Note that this means agents may lie even worse about themselves than they actually are (while “lying positively” about themselves), in case their current reputation in the eyes of the receiver (or rather their estimation thereof) is very low.

Additionally, as mentioned above, agents run the risk of accidentally revealing their lie by blushing whenever they lie. This happens with a fixed probability of 10%, that is, on average with every tenth lie.

### 2.3 Agent strategies

The above process describes how so‐called *ordinary agents* communicate in the RGS, where the choices of conversation partner and topic happen randomly and where lies are positive when about themselves and about their friends, but negative when about enemies. The same holds for *deceptive agents*, whose only difference is that their honesty is set to 0% (but who are otherwise like ordinary agents, thus lacking a specialized communication strategy). Additionally, there are three special strategies, shown to introduce interesting dynamics in a previous work (Enßlin et al., 2022). All special strategies have an intrinsic honesty of 0 (which is why we might refer to all of them as “deceptive” in the following work, as opposed to the “deceptive strategy” that explicitly refers to the just introduced deceptive agent), and they use more sophisticated policies in order to boost their own reputations. Specifically, they do so by strategically choosing conversation partners and topics whenever they can, that is, whenever initializing a conversation. *Manipulative agents* preferentially talk to agents they believe to be dishonest. Thus, the probability of choosing an agent as conversation partner is proportional to $1-{\bar{x}}_{ab}$, where ${\bar{x}}_{ab}$ is the mean of the manipulative agent a’s current opinion of how honest the potential conversation partner b is. Further, when choosing the topic of conversation, they always choose their conversation partner. Whenever manipulative agents then talk about their conversation partners, they flatter, or lie positively. Given the aforementioned link between friendship and reputational messages, this means that manipulative agents befriend their conversation partners effectively, and in turn, encourage them to lie positively about themselves in any later conversation to others. *Dominant agents*, in contrast, prefer to talk to honest agents. Thus, the probability of choosing an agent as conversation partner is proportional to ${\bar{x}}_{ab}$, where ${\bar{x}}_{ab}$ is the mean of the dominant agent a’s current opinion of how honest the potential conversation partner b is. Further, they like to talk about themselves, but as learning about the other agents is also important, dominant agents do not always choose themselves as topic but only in half of the cases. In the other half, the topic is chosen randomly, same as an ordinary agent would do. In every conversation where the dominant agent is the topic, it lies positively, boasting its own honesty. If their conversation partners believe those lies, they will further distribute that message as their own, true belief in further conversations. Finally, *destructive agents* do not try to increase their reputation in any way, but instead, decrease the reputations of their enemies. Thus, destructive agents also preferentially talk to honest agents (same as for dominant agents), but always choose one of their enemies as conversation topics in order to badmouth them, thus lying negatively. To compensate for not promoting themselves, destructive agents never blush when lying, which helps them to lie without being detected. We will later use one of these special agents among two ordinary agents to study the effects of their conversation strategies on the social dynamics in the 3‐agent system.

### 2.4 Learning

After both conversation partners have communicated their messages about the same topic, they have to interpret the received information and draw conclusions as best they can. In the easiest case, the agents would just observe whether their conversation partner has lied or not, and thus update their beliefs based on the number of honest and dishonest statements that agent makes. An example of such an evolution when the observed agent is honest in 80% of the cases is shown in Fig. 1, where the more data the agents gather, the steeper and stronger their opinions grow.

In general, however, it is not a priori clear whether or not the speaker lied, so the receiver typically has to consider both options, usually weighted with different probabilities. Furthermore, as the topic of conversation could be the reputation of any agent (not just the conversation partners), agents will often have to simultaneously update their beliefs about the honesty of their conversation partner and about some third agent based on the same message.

Both updates would thus usually result in superpositions of possible belief states, which rapidly becomes very complex after a few rounds of conversations. To deal with that complexity, and also to mimic the imperfection of human memory and the shortcuts it uses (Fiske & Taylor, 1991; Simon, 1955; Tversky & Kahneman, 1974), agents compress that complex belief state back into a single beta distribution for each other agent separately. This set of one‐dimensional beta distribution parameters is then memorized by the agents after every update, instead of the complex, mathematically correct multi‐dimensional belief state that would capture the entanglements of the agent's honesties, with reference to each other.

By minimizing the amount of information that is lost during this step, we ensure that the agents still extract as much information as possible from the available data.^5^ The omission of such entanglement between the knowledge on the different aspects has been shown to lead to various kinds of confirmation biases (Pilgrim, Sanborn, Malthouse, & Hills, 2024). This was already discussed in the initial publication on the RGS (Enßlin et al., 2022).

### 2.5 Learning cues to credibility

A final, crucial aspect of belief updating is how agents should judge the credibility of the received messages in the first place: How do they know to what degree they should believe the speaker and trust the message's content? The agents use two measures for this decision: The overall honesty they currently assign to the speaker, and the ratio between how likely the observed data is given a dishonest statement compared to an honest statement. The exact formula how both measures are combined is given in Appendix A.2. Let us focus on the latter likelihood ratio, though, as the observed data includes the blush cue, which is crucial for the LICS as we go on to show.

For this likelihood ratio, agents use three^6^ criteria (how they are combined is shown in the Appendix, Eq. A.4): how surprising the received message was; whether or not the speaker made a confession; and whether or not the speaker blushed. Surprise, measured by the KL Divergence between the incoming message and the receiver's previous knowledge, is used to make the agents skeptical if the messages deviate too much from their current worldview. The higher the surprise of a received message, the less likely it is to be perceived as honest (see footnote 4). This prevents the agents from believing arbitrarily large lies that would have huge impacts, thus forcing the liars to keep their lies realistic (and small). Confessions are used, on the other hand, to detect situations in which the speaker should be definitely believed: Whenever a speaker talks about itself communicating a worse opinion than what the receiver already believes, this statement should be believed. If it were a lie, the speaker would have tried to communicate a better image of itself. Even if the statement indeed was a lie and the speaker just misjudged the receiver's opinion, it is still reasonable to update according to the message as it lowers the liar's reputation. On the third criteria—whether not the speaker blushed—we want to focus on in a bit more detail: As we (from an outside perspective) know, the probability of blushing for a dishonest statement is $f_{b|\neg h}=10\%$ and the probability of blushing for an honest statement is $f_{b|h}=0\%$, we can conclude that, whenever the speaker blushed, this statement was a lie. On the other side, if the speaker did not blush, we can not directly infer the honesty of this message, but it is a hint toward a higher credibility. More generally, we can formulate a ratio between the likelihoods of observing a blushing sign in dishonest compared to honest statements

(4) $$\begin{array}{c} R\left(\mathrm{blush}\right)=\frac{P(\mathrm{blush}|\neg h)}{P(\mathrm{blush}|h)}=\frac{f_{b|\neg h}}{f_{b|h}}, \end{array}$$

which should ideally be R(blush)=0.1/0=∞. Thus, if agents inherently knew the correct blushing frequency, they would always correctly infer that any message with a blush is a lie. However, in the dual‐learning problem characterizing these simulations, agents do not know this frequency. Rather, they must simultaneously learn it and all agents' honesty levels by observing co‐occurrences of blushing and reputational messages, which both involve uncertainty.^7^

Naturally, this method does not lead to perfect knowledge, but perfection is also not necessary in order to draw the right conclusions. Since (apart from being not the only criterion for lie detection) only the ratio $f_{b|\neg h}/f_{b|h}$ (Eq. 4) is used in the agents' belief updating, it is sufficient if the two estimated frequencies are different enough to produce a reasonably large ratio. Though not infinite, it will often be high enough for agents to associate blushing signs with dishonest statements in the vast majority of cases. If, however, the estimated blushing frequencies become too similar, R(blush) becomes close to 1 and the agents would effectively ignore any observed blushing signs in their belief updating. In other words, they would come to think that blushing is equally likely in dishonest and in honest statements, and would consequentially not assign it any evidential value. This state, which emerges from agents' experiences, is what we call a Learned Insignificance of Credibility Signs (LICS) bias. To be precise, we consider an LICS not only the exact equality of both frequencies but allow for an interval of 20%, defining LICS to be cases where $1.1f_{b|h}>0.9f_{b|\neg h}$. This already reduces the evidential value of a blush cue in the agents' belief updating significantly. A more detailed explanation of how agents assessed the credibility of received messages and of their corresponding belief updating, including about blush cues, is given in Appendix A, and a first analysis of the agents' learning success can be found in the Supplementary Material. The code supporting this study is openly accessible at RGS.

## 3 Results

The results are divided into two kinds of simulation runs, described in the introduction. First (Section 3.1), we concentrate on learning runs: how agents learn about blush cues and under which circumstances an LICS emerges. The second (Section 3.2) focuses on rehabilitation runs, wherein we place agents that developed an LICS during learning in new social contexts, examining whether it is possible to escape from an LICS and which consequences this has for the agents' worldviews.

### 3.1 Learning runs: The emergence of a LICS

#### 3.1.1 Observations

We examine agents' learning of blushing frequencies $f_{b|\neg h}$ and $f_{b|h}$. First, to establish that agents are, in principle, able to learn the correct frequencies, we use a setup with three ordinary agents, comparing the basic case, wherein agents inherently know the exact frequencies ($f_{b|\neg h}=0.1$, $f_{b|h}=0$) with the more complex dual‐learning problem that motivates the present study, wherein agents must infer those frequencies while simultaneously learning about others' intrinsic honesty.

**Fig. 2:**
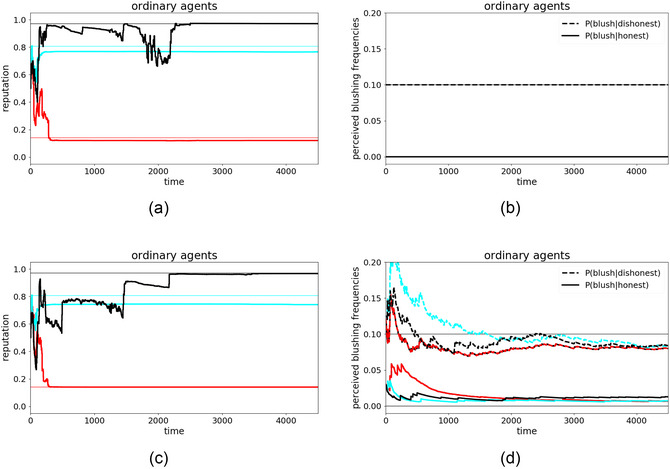
Comparison of reputation dynamics where the agents either know the exact blushing frequencies (a, b, or top) or have to learn them (c, d, or bottom), showing both the evolution of agents' mutual reputations (a, c, or left) and estimates of blushing frequencies (b, d, or right). The reputations show the average honesty of an agent as perceived by others, while the thin lines show the agents' intrinsic honesties (i.e., the ground truth) in the agents' respective colors. For blush frequencies, dashed lines indicate the evolution of $f_{b|\neg h}$ and solid lines indicate the evolution of $f_{b|h}$. The color of the lines represents the color of the agent whose estimate it is. In (b), the agents know the exact values, such that there is no evolution, and the perceived blushing frequencies are constantly at the correct value. In (d), however, we see the agents' learning progress over time and that their perception converges toward values not far from the ground truths at $f_{b|\neg h}=10\%$ and $f_{b|h}=0\%$ (thin black lines).

**Fig. 3:**
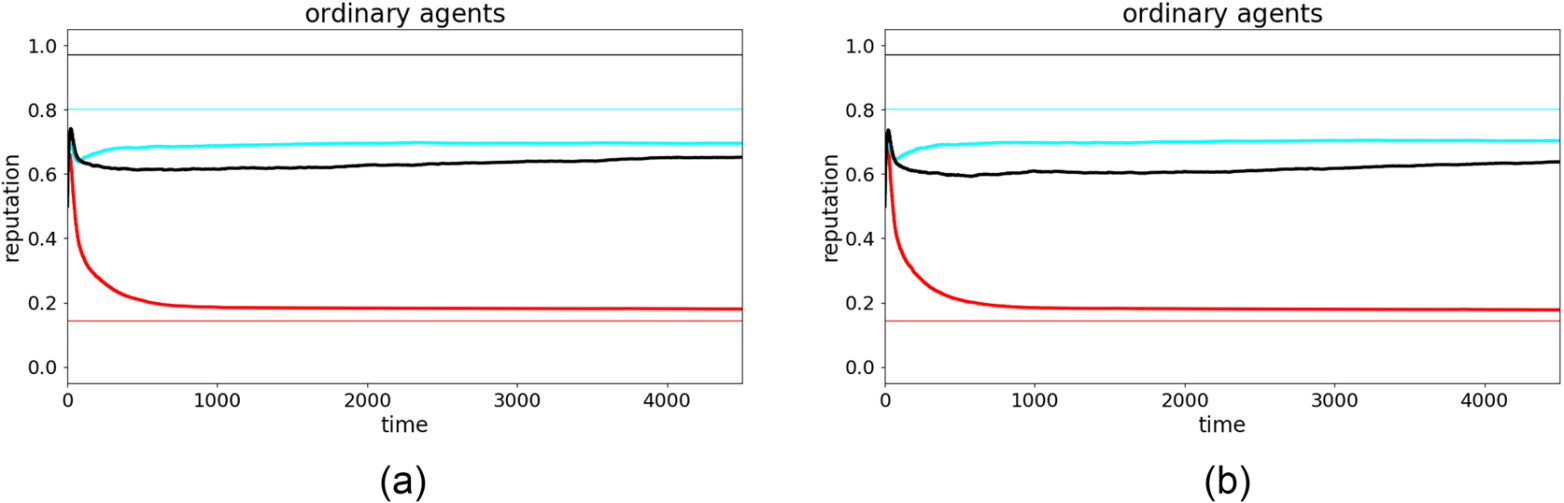
Statistics of reputation dynamics. The lines (color code as before) show the average evolution of mutual reputations over 1000 simulations with varying random seeds. (a) Results from the basic setup where the exact blushing frequencies were given to the agents. (b) Results from the dual‐learning task, where the blushing frequencies have to be inferred from the agents' experiences throughout the simulation. Agent red's and cyan's reputation match their honesty in both types of simulations, while agent black's reputation is, on average, lower than its honesty deserves, again consistent in both simulations.

Although ordinary agents do not use special deception strategies, they can still vary in intrinsic honesty. For this proof‐of‐concept, the simulations include three ordinary agents with intrinsic honesties of 97% (black), 80% (cyan), and 14% (red).^8^ Thus, over half of the messages (64%) in the system will be honest on average, and the other 36% will be lies. Fig. 2 provides two illustrative single runs where the agents are either informed about the exact blushing frequency (top) or have to infer it (bottom); Fig. 3 shows the overall pattern across 1000 runs of each type.

In both illustrative single runs, agent black's honesty was eventually correctly estimated by the other agents, as was agent red's honesty (Fig. 2a,c). Agent cyan was slightly underestimated, but the assessments were still reasonably accurate, suggesting that agents succeeded well at this aspect of the dual‐learning problem. Agents also managed the additional task of inferring the blushing frequencies (Fig. 2b,d), where the learnt frequencies are close to the known ones (as mentioned, given the role of the likelihood ratio in belief updating, the exact value of the blushing frequencies matters less than their distinctiveness, which is clearly visible in Fig. 2). Despite being faced with a substantial proportion of lies, we can see that it is, in principle, possible for agents to learn the correct frequencies.

Both illustrative runs were typical of these proof‐of‐concept simulations more generally. Fig. 3 shows summaries from 1000 simulations for the honesty estimates in the basic task (a) and the dual‐learning task (b). As the reputation dynamics are strikingly similar, we conclude that the agents are generally able to infer the blushing frequencies well, or at least well enough to form the same opinions as before despite the complexity of the dual‐learning task.

**Fig. 4:**
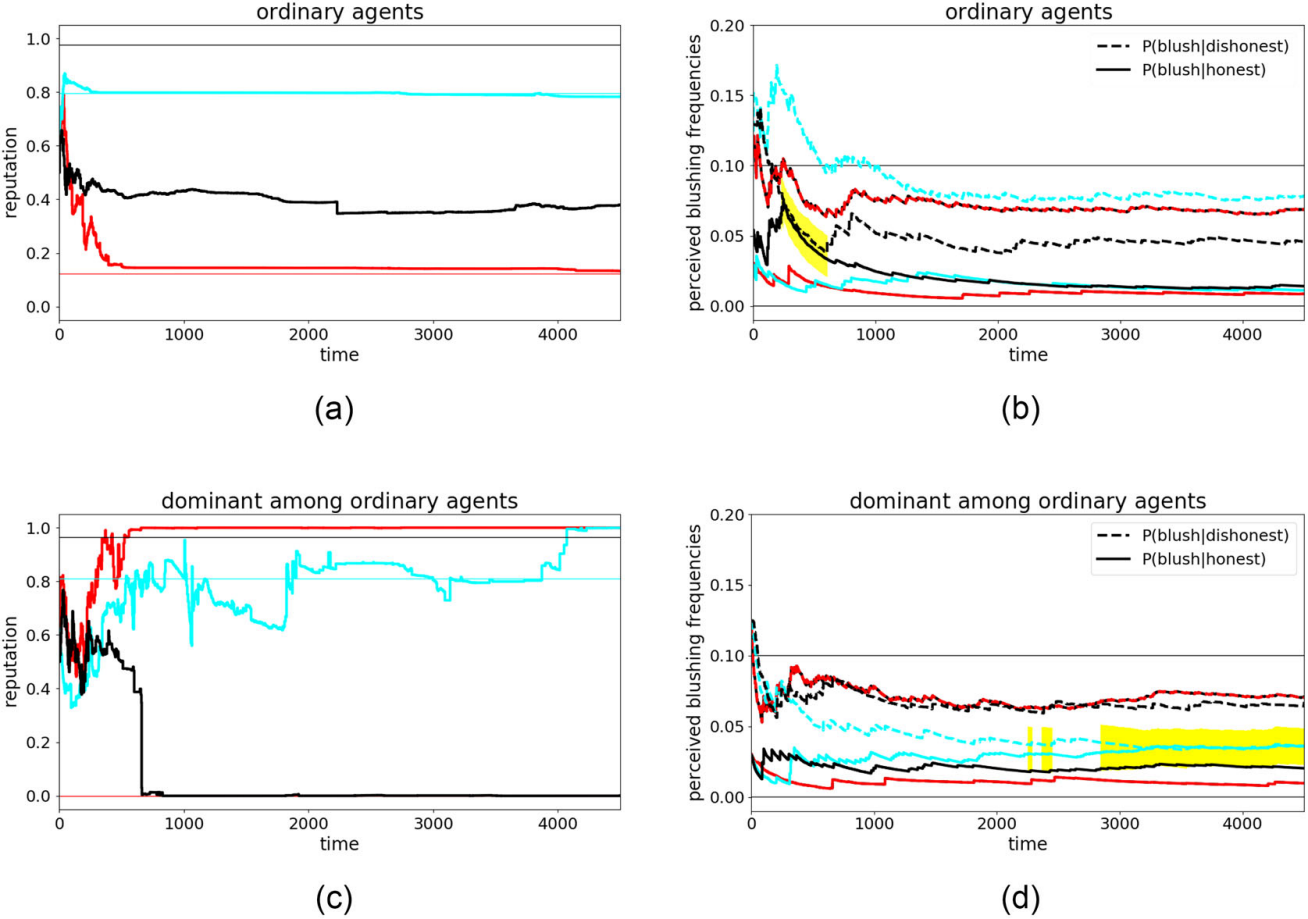
Exemplary simulations to demonstrate the emergence of an LICS. The color code is the same as in Fig. 2. The top panels illustrate a simulation run with three ordinary agents, showing (a) the reputation dynamics; and (b) the corresponding dynamics of the blushing frequencies. In this run, agent black gets confused w.r.t the blushing frequencies at the beginning of the simulation (approx. times 200–600, yellow highlighted area in (b)). Here, black's perceived blushing frequencies become very close, indicating an LICS during this time interval. The bottom panels illustrate a similar run, but with agent red playing the dominant strategy, again showing (c) reputation dynamics, where the reputations of agents red and black are wildly misestimated by the others; and (d) blushing frequencies, where agent cyan develops an LICS first around time 2200, from which, it does not recover during the rest of the simulation (yellow highlighted area in (d)).

Although agents generally succeeded at the task, we also observed individual runs where they failed, where either chance or external circumstances lead to wrong estimations of the blushing frequencies. Two such runs in which the agents (partially) failed in the dual‐learning task are shown in Fig. 4.

In the upper panels, we see three ordinary agents (the exact same setup as in Fig. 2 but with a different random seed). In Fig. 4a, we observe that agent cyan's and red's honesties were estimated accurately, whereas agent black was severely underestimated by both other agents. Despite its intrinsic honesty of 97%, its reputation ends up around 40%. Such dynamics have been seen in previous works already, referring to it as the RGS‐version of the Cassandra Syndrome (Enßlin et al., 2022).

For the same case, agent black's estimation of the two blushing frequencies was almost equal near the beginning of the simulation (yellow highlighted area, Fig. 4b). We classify this as an LICS as $1.1f_{b|h}>0.9f_{b|\neg h}$. However, since agent black's opinions of both cyan and red were still fairly accurate, agent black was soon able to recover from its LICS all by itself. This spontaneous recovery from an LICS is very common in simulations with three ordinary agents, but the dynamics change as soon as we introduce special strategies, which were always deceptive, and which had additional nonepistemic motives.

One case where the emergence of an LICS is particularly clear is when the group includes a dominant agent. The lower panels of Fig. 4 show an illustrative simulation run with a dominant agent red among two ordinary agents cyan and black. From previous works (Enßlin et al., 2022), we know that a dominant agent often causes a chaotic phase at the beginning of the simulation, from which, it frequently emerges as very highly reputed (as soon as an RGS‐version of narcissistic supply sets in). This pattern can also be observed here in Fig. 4c, before timestep 1000. The opinion dynamics then usually freeze and the delusion‐like state of both other agents persists, with red remaining highly reputed for the rest of the simulation (from time‐step 1000 onward). Moreover, we see that red and cyan's perceptions of agent black's reputation was destroyed during the chaotic phase and finally froze at a very low value. This is often the case and relates directly to the dominant strategy: As agent red mainly focuses its propaganda on the most honest agent, that is, black, the latter usually develops a positive opinion about red first. As soon as black communicates that to agent cyan, it is typically regarded as a liar as agent cyan still has a bad opinion on agent red. Only after some time, agent cyan is convinced of agent red's high honesty, but agent black's low reputation is already settled at this point. In the end, agent red has a high reputation, while agent black is perceived as liar, first by cyan and later also by red. Consequentially, agent cyan ends up with a completely inverted worldview: is believes red to be very honest and black to be a liar. At the same time, cyan observed red blushing regularly, while the apparent lies from black were never accompanied by blushes. Thus, cyan learned to assign very little evidential value to blush cues: Cyan's $f_{b|\neg h}$ and $f_{b|h}$ evolve to become very similar, constituting an LICS (yellow highlighted area in Fig. 4d). Thus, by the end of the run, cyan has learned a bias against disconfirmatory evidence in that it completely discounts objectively reliable signals that its beliefs are false. We probe the more general sociocognitive causes of this delusion‐like state in the next subsection.

**Fig. 5:**
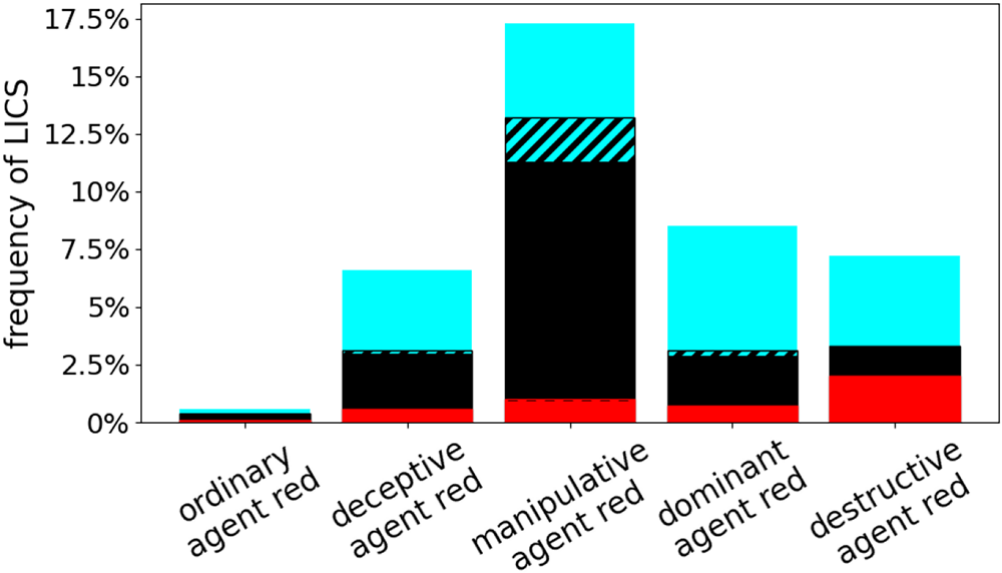
Statistics of the emerged LICSs for different strategies played by agent red. For 1000 simulations with different random seeds, the percentage of simulations where at least one of the agents developed an LICS is shown. The distribution of the frequencies among the three agents is shown in their respective colors. The x‐axis indicates the strategy played by agent red, while agents cyan and black are always ordinary agents. Note that in some simulations, more than one agent develops an LICS (striped areas).

Fig. 5 summarizes the number of times an LICS emerged across 1000 simulations with 4500 timesteps each, where agents blue and cyan were always ordinary and agent red had either an ordinary, deceptive, or a special strategy. The temporary LICS case for three ordinary agents (cf. Fig. 4a,b) turns out to be rare overall, but an honesty of 0 and the use of a special strategy caused an emergent LICS phenomenon in a substantial minority of runs. We see that the transition from a mostly dishonest agent (14%) to a completely dishonest agent (0%) makes a clear difference. As we will see later in Section 3.1.3, this can not only be related to the higher amount of misinformation in the system, but is mainly caused by the absence of confessions and the inability of ordinary agents to guess the deceptive agent's true beliefs, which elevates the latter to a more powerful position when it comes to influencing the ordinaries' beliefs (Kainz et al., 2022). We should note that this is exactly the reason why all other special strategies were designed to be fully deceptive in the first place. As the deceptive agent does not use any specific communication strategy and targets both ordinary agents equally, it is also not surprising that the fraction of both ordinary agents falling for an LICS in the presence of a deceptive agent is pretty balanced. This is not the case for the other special strategies. Both the pure frequency of LICS and the asymmetry between the two ordinary agents is highest when agent red was manipulative: The manipulative strategy turned out to reliably establish an incorrect perception of others agents' worldviews, consistent with previous work even without the dual‐learning problem (Enßlin et al., 2022). As the manipulative agent mainly targets the less‐honest agent cyan, converting it into a friend that lies positively about red, agent cyan itself is not affected that much (opinion‐wise). However, agent black feels the consequence of having both other agents praising red whenever there is the opportunity, which often makes agent black believe their propaganda. Together with the frequent blushes black receives from red, its confusion is predictable with the logical consequence that it will more likely fall for an LICS.

In contrast, the dominant strategy follows a high‐risk/high‐gain approach. It causes a chaotic phase (cf. Fig. 4c) in about half of all simulations, later freezing in with a specific set of opinions (Enßlin et al., 2022). These can include a very high reputation of the dominant agent if the ordinary agents happen to believe its self‐propaganda (as in this example run). However, if the ordinary agents detect the deception, there is also the chance for the dominant agent to fail completely. Consequently, the risk of falling for an LICS in the presence of a dominant agent is generally reduced compared to the very reliable manipulative strategy. It is especially lower for agent black, who is usually not that strongly affected by the dominant agent's propaganda compared to the less‐honest agent cyan, as seen in the example above. Although the frequency of an LICS under a dominant agent is not significantly higher than under a deceptive agent, the reason for LICS is quite different as just explained. The two scenarios of either succeeding or failing are also well visible in the distribution of perceived blushing likelihood ratios shown in the Supplementary Material. The destructive agent is the least effective strategy in terms of gaining high reputation as it does not even try to manipulate the others in such a direction, but instead focuses on bad‐mouthing enemies. Accordingly, the frequency of emergent LICS in the presence of a destructive agent is even smaller than for a dominant agent. Moreover, we see that the destructive agent suffers from LICS quite often itself, which lowers its ability to interpret and judge the others, making it even harder to influence them in any direction. This is caused by the fact that destructive agents do not blush when lying, and consequentially, lack the reliable information from self‐perception that other agents have as part of their data on blushing. What was previously intended to compensate for the non‐self‐promoting communication strategy seems to be an even larger disadvantage in this dual‐learning problem. Still, we see an asymmetry between the effects on ordinary agents black and cyan, which is caused by the fact that the destructive agent red badmouths its enemies, usually agent black (as agent cyan is more often able to befriend red given its more frequent lies). Thus, agent black is usually less reputed than it should be, while at the same time, agent red is usually a lot higher reputed than it deserves, which leads agent cyan to disregard the evidential value of red's frequent blushes and black's rare blushes.

As one might have deduced from these examples already, there are many possible causes and circumstances that can lead to an LICS. The connections are complex and vary depending on the specific communication strategies. Nevertheless, there are two essential quantities to which all of this can be traced back: the reputations the agents assign to each other and their informednesses about the environment. The following subsection explores these dynamics in more detail while investigating the general causes and effects of the LICS in the RGS.

#### 3.1.2 Causes and effects

As established above, an LICS emerges in the RGS when agents learn to discount the evidential value of the blush cue, ultimately meaning that they display a bias against disconfirming evidence such that objective cues of falsehood do not perturb their previously held beliefs.

It seems driven by both the low honesty and the use of special communication strategies by agent red, which is unsurprising as both enable agent red to effectively manipulate the others' opinions and convince them of its own high reputation. In reality, red agent's honesty is 0, meaning it blushes approximately every tenth statement, which conflicts with its goal of appearing honest. However, if agent red manages to convince the ordinary agents that blushing signs do not have any evidential value for their lie detection, it becomes much simpler for agent red to achieve and maintain a high reputation.

**Fig. 6:**
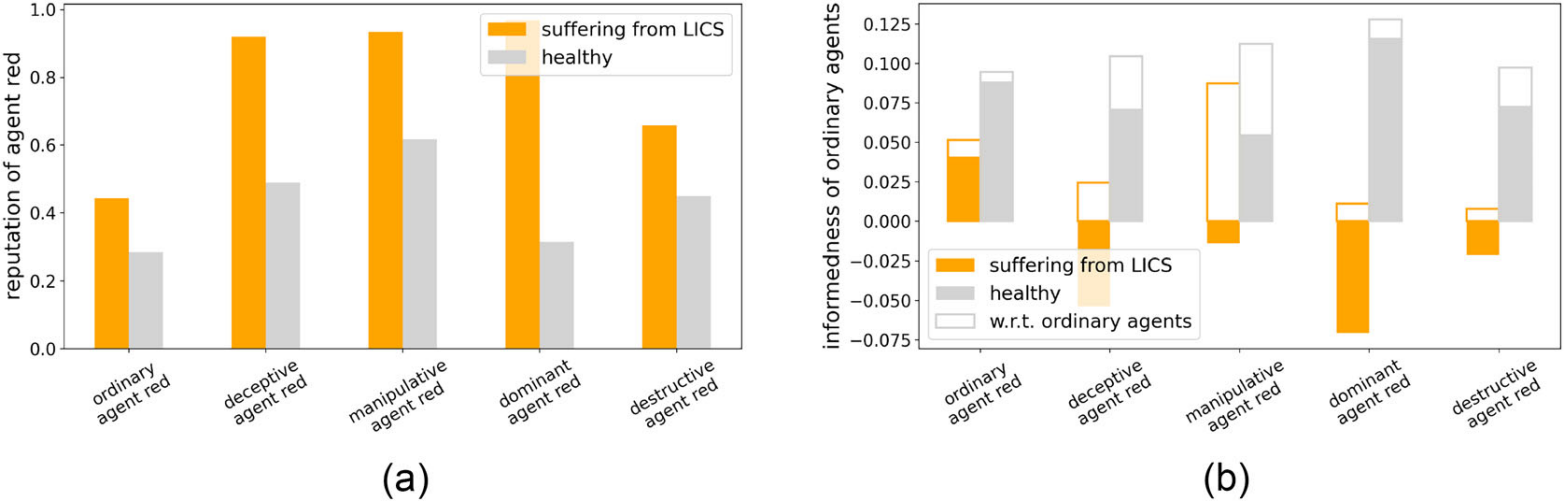
The influence of an LICS on reputation and informedness. (a) The average reputation of agent red that is held by the agent suffering from an LICS (orange) or the same agent at the same time in a different simulation where it did not suffer from an LICS (gray). (b) The average informedness of ordinary agents while suffering/not suffering from an LICS. Filled bars indicate the informedness w.r.t. to all agents, that is, the informedness about the deceptive agent, themselves, and the other ordinary agent, while nonfilled bars show the informedness about ordinary agents only, that is, about themselves and the other ordinary agent. In both panels, a comparison between the different strategies used by agent red is shown. Note: The average is not only taken over times where an LICS was present, but also including the preceding 500 timesteps in order to account for effects that usher in an LICS state.

This implies that two quantities should be especially related to the occurrence of an LICS: the reputation of agent red (which should be higher whenever the other agents suffer from an LICS) and the other agents' informedness.^9^ As informedness is an index of how accurate their representation of the world is, it should be lower when they suffer from an LICS. We see these expected patterns, both for red's reputation and for other agents' informedness, in the simulation outcomes (Fig. 6).

Starting with agent red's reputation, Fig. 6a shows that the higher an ordinary agent's estimation of agent red's honesty, the higher that agent's chance is of suffering from LICS. The causal direction of this effect, however, is less clear. A high reputation generally brings more influence on others' opinions as reputation in the RGS is equal to credibility. This makes the lies of deceptive agents more powerful and others will incorporate the lies' content into their knowledge. As those lies typically are in favor of the deceptive agents, their high reputation gets further amplified. Not only is this a self‐reinforcing effect, but also one that causes misinterpretation of blush cues in the ordinary agents' minds. As soon as deceptive agents are highly reputed in others' minds, the latter will downgrade the evidential value of blush cues coming from the deceptive agents, leading to an LICS. At the same time, an already existing LICS causes ordinary agents to misclassify the deceptive agents' blushing signs, which, in turn, increases (or at least, avoids decreasing) the reputation of the deceptive agent. Thus, a downward spiral emerges with highly reputed deceptive agents and misled ordinary agents.

Agents' informedness is closely intertwined with this downward spiral: By definition, perceiving a deceptive agent as having a high reputation already means one is misinformed. This pattern, too, is obvious in the simulation outcomes (Fig. 6b). Informednesses was lower under the influence of an LICS compared to cases where an ordinary agent correctly estimated the blushing frequencies.

Fig. 6b shows how ordinary agents have lower informedness about all ordinary agents (including themselves^10^) when the agent suffers from an LICS. This implies that an LICS not only distorts the affected ordinary agent's view of the deceptive source, but also their perception of the rest of their environment. Again, we can observe a similarly self‐reinforcing effect as before: Being poorly informed (especially about a deceptive agent, but also about the rest of the system) leads to wrong judgments of the received messages and thus misclassifications of blushing signs, yielding an emergent LICS. Conversely, an LICS hinders the suffering agents from correctly judging the meaning of blushing signs, leading to worse lie‐detection, and thereby, worse inferences about reputational messages, increasing misinformedness.

Both spirals thus go hand‐in‐hand and are schematically illustrated in Fig. 8a. This suggests a major difference between previous simulations without the possibility of an LICS (when agents inherently knew the blushing frequency) and the new setup (where they face a dual‐learning problem): A new mechanism in the dual‐learning case allows deceptive agents to stabilize their high reputations more efficiently.

**Fig. 7:**
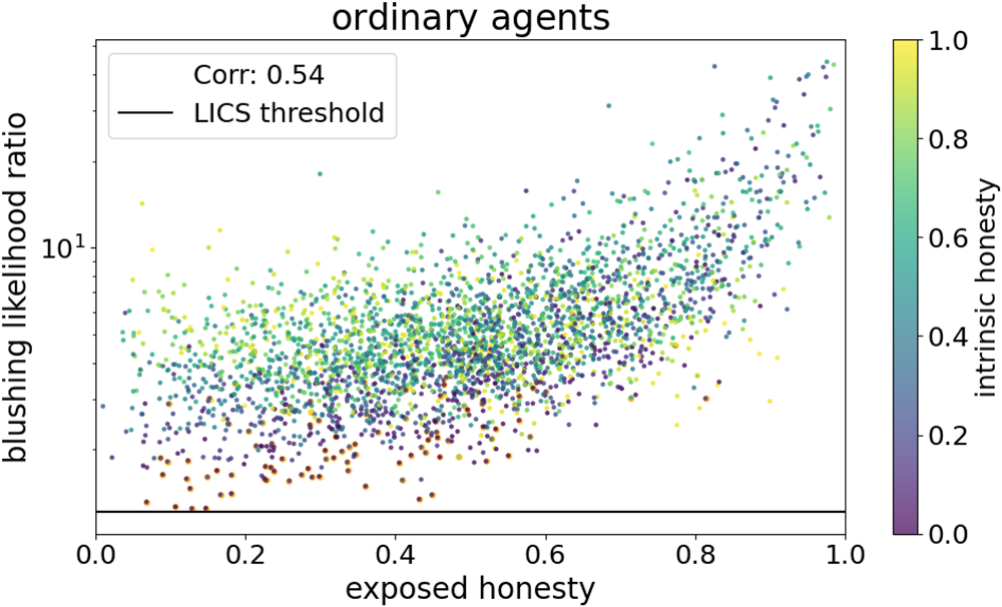
Correlation between the honesty an agent is exposed to and the time‐average of its estimation of the blushing likelihood ratio in 1000 simulations with three ordinary agents and uniformly distributed intrinsic honesty values. The exposed honesty is the average honesty of all other agents in the system (except for the agent's own intrinsic honesty, which is shown by color). Furthermore, the threshold of LICS at 11/9≈1.22 is shown. Note, though, that the dots are time‐averages, that is, some agents did suffer from LICS temporarily in the simulation, although the dot is not below the threshold. All cases, where an agent suffered from LICS for at least one timestep, are highlighted by an orange circle around that agent's corresponding dot, mostly located at low blushing likelihood averages.

**Fig. 8:**
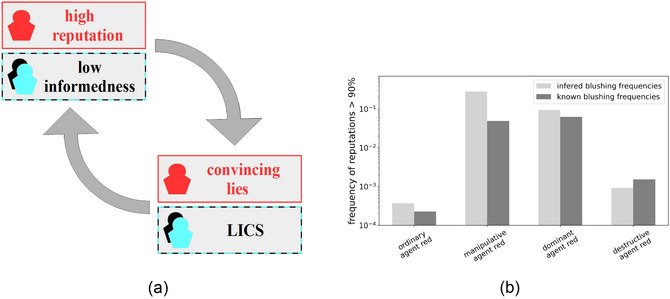
(a) Schematic representation of the mechanism that enables deceptive agents to stabilize their high reputation. As soon as a deceptive agent is very well‐reputed by other agents, the latter are less informed by definition. Once such a state is established, a self‐amplifying effect maintains it: The deceptive agent's lies will be believed more frequently as the others already have a positively biased estimate of its honesty. At the same time, ordinary agents still observe many blushing signs from the deceptive agent, which leads the ordinaries to wrongly infer that blushing can occur with honest statements, reducing its evidential value as a cue of lying. In other words, they learned the insignificance of blushing as a credibility sign. Furthermore, this LICS hinders the ordinary agents from correctly judging the next received statements, resulting in an even lower informedness. Especially w.r.t. the deceptive agents, whose blushing signs have now even less significance to the ordinary agents, their LICS offers the best opportunity to continue successfully sowing lies in its own favor. (b) Comparison of reputation statistics on a logarithmic scale between simulations where the agents have to infer the blushing frequencies from their experiences and simulations where the exact blushing frequencies are known to the agents. We focus here on top reputations, that is, show the frequencies with which the special agents (see individual strategies on the x‐axis) achieve reputations of 90% or higher. Note that the ordinary agent here has an intrinsic honesty of 14%, while all others are fully deceptive (have an intrinsic honesty of 0%).

The effect of this is shown in Fig. 8b, which compares the frequency of top‐reputed special agents with and without the dual‐learning problem. 3 out of 4 special strategies (deceptive, manipulative, and dominant) achieve top reputations of 90% or higher more often when agents have to infer the blushing frequencies compared to when they know the exact values. An LICS (including also weaker confusions of blushing frequencies prior to the emergence of full‐blown LICS) helps deceptive agents to achieve very high reputations and to stabilize them. The only exception is the destructive agent: It seems that the new additional task of inferring blushing frequencies did not help—and indeed, may have prevented—destructive agents from achieving top reputations. This is because the destructive agent is the only one that suffers from an LICS itself in a significant number of times (see Fig. 5), which hinders the destructive agent's ability to assess and influence reputation accurately, including its own (cf. Fig. 5). 

A deeper investigation of this hypothesis, including emerging correlations of the discussed parameters, is provided in the Supplementary Material, along with a discussion of how deceptive agents achieve high reputations in the first place.

All in all, the additional task of inferring blushing frequencies in the dual‐learning version of the RGS boosts the likelihood of developing an LICS, simultaneously making it harder for ordinary agents to develop a correct worldview once they have fallen for deceptive strategies and making the deceptive strategies more effective.

#### 3.1.3 Influence of honesty on LICS

One might argue now, that this simulation setup—three agents being 14% (0%), 80%, and 97% honest—is a very specific choice. We thus want to investigate the influence of the agents' intrinsic honesty values on the emergence of LICS in general. For this, we use a setup with three ordinary agents with uniformly distributed honesties. After drawing those honesties, we sort the agents by color, where agent red still is the least honest agent in each simulation (thus implying an average honesty of 25%), agent cyan is in the middle (with an average honesty of 50%), and agent black is the most honest agent (75% on average). Again running 1000 simulations with varying random seeds, we can observe how the amount of misinformation influences the agents' ability to correctly calibrate the blushing likelihood ratio R(blush) (Eq. 4). In Fig. 7, we see a clear correlation between the honesty an agent is exposed to (i.e., the average honesty of the other two surrounding agents) and its estimated likelihood ratio. Thus, being in an honest environment helps the agents estimating the significance of blushing signs. At the same time, we see that even in very dishonest environments (those with less than 20% honest statements), the agents usually estimate the ratio to be between 2 and 7, which already yields a significant bias in their reasoning toward lower message credibility when accompanied by a blushing sign. Nevertheless, the risk of falling for an LICS (at least temporarily) is still a lot higher when an agent's environment is less honest, especially if the focal agent itself is very dishonest, too. For honest agents, the estimated blushing frequency is usually a bit higher (4–8) even in those extremely dishonest environments.

When relating this to the observation above in Fig. 5 that agent red's transition from 14% honesty to 0% honesty causes a large difference in the frequency of emergent LICS, we can now be sure that this is not only due to the amount of misinformation in the system as a whole. For agents black and cyan, this switch from ordinary agent red to deceptive yields a difference in exposed honesty from 55.5% to 48.5% or 47% to 40%, respectively, which does not change too much in the statistics of estimated blushing likelihood ratios (Fig. 7). Rather, the main difference is the concentration of the full dishonesty in a single agent that neither makes any confessions nor reveals anything else of its true worldview, as discussed above.

### 3.2 Rehabilitation runs: Unlearning an LICS

Finally, in hopes of a solution to the problem of emergent LICS (and the knock‐on social problems outlined in the introduction), we aim to explore how compromised agents might recover from an LICS. Especially in the vicinity of a special agent, this can be difficult, as Fig. 4d demonstrates. There, agent cyan developed an LICS under the influence of the dominant agent red. The LICS was stable over the second half of the simulation. As agent cyan's LICS strengthened with each further blushing sign it received from agent red that it classified incorrectly, a spontaneous recovery without external assistance seems unlikely.

Instead, we investigate the possibility of removing such an agent from the toxic social environment in which the LICS was developed, seeing whether it can recover from its persistent devaluation of cues to credibility in some other environment. For this, we use the following simulation setup: We take agent cyan with its complete internal state (beliefs about reputations and about blushing frequencies at the end of the simulation shown in Fig. 4c,d) and insert it in a new environment with new ordinary agents green and yellow.

Though they lack special communication strategies, these new agents do not have to be completely honest,^11^ so we assign the new agents random intrinsic honesties between 0 and 1, resulting in agent cyan being exposed to an average honesty of 0.5 (or 50%). This setup resembles a system at an imaginary vertical line at 0.5 in Fig. 7, showing that we do not rely on especially honest environments where developing an LICS is fairly impossible (right third), but use a mid‐honesty‐regime. Note that the average honesty in the system is also very close to the value it previously had, when agent cyan developed the LICS under the influence of the dominant agent red. Previously, agent cyan was exposed to (0%+97%)/2=48.5% honest statements, which is close to the 50% in the rehabilitation runs. The two main differences between the learning and rehabilitation runs are thus: (1) the distribution of dishonesty across both ordinary agents green and yellow (instead of having one fully deceptive and one very honest agent); and (2) the absence of a dominant communication strategy.

**Fig. 9:**
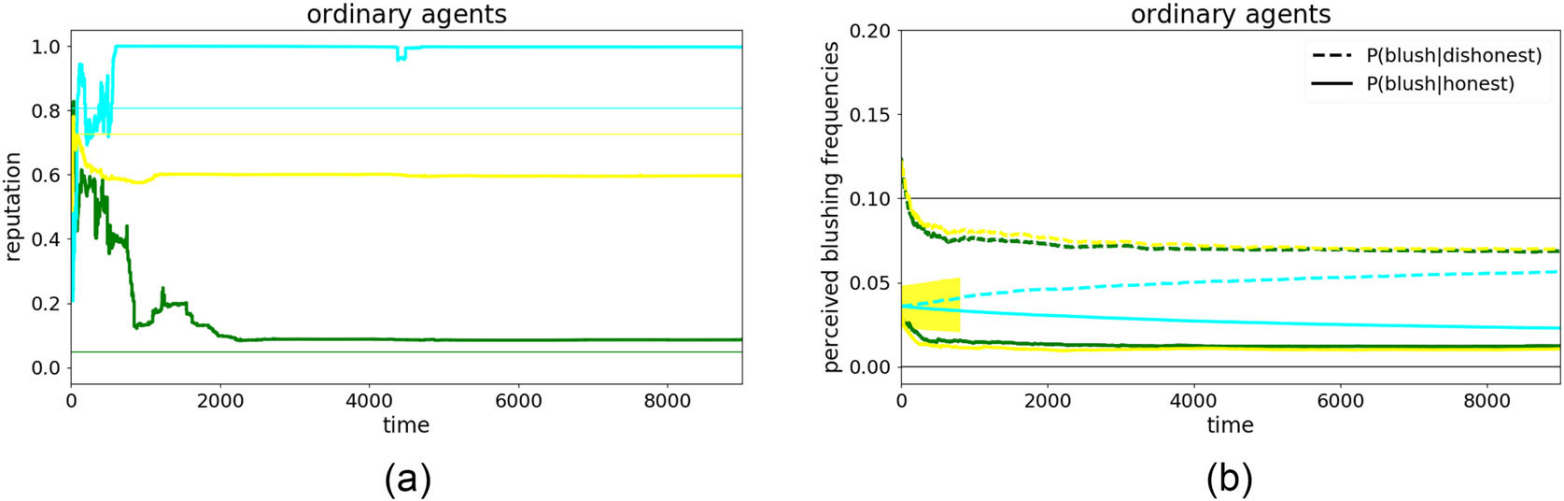
Evolution of agent cyan in a new environment, consisting of two ordinary agents yellow and green. (a) Illustrative simulation of all agents' mutual reputations; (b) average evolution of the agents' perceived blushing frequencies across 20 simulations with varying random seeds. Agents yellow and green also have different intrinsic honesties in each simulation to demonstrate that the recovery of perceived blushing frequencies does not rely on the surrounding agents' honesties.

With this setup, we ran 20 simulations with different random seeds. Fig. 9a provides an illustrative example of reputation dynamics in one such simulation run,^12^ while Fig. 9b provides a summary of estimated blushing frequencies across multiple such runs.

The reputation dynamics in Fig. 9a show a typical pattern, wherein the honesty frequencies of all agents are ultimately perceived reasonably, although in this run, agent cyan's honesty was overestimated and yellow's underestimated by both other agents. Such a scenario is common among ordinary agents and can be understood as healthy. In Fig. 9b, we can observe healthy rehabilitation dynamics with respect to the agents' perceived blushing frequencies. Agent green's and yellow's perception of both frequencies are consistently distinct (as usual for ordinary agents in nondeceptive environments), and agent cyan's perception recovers from its previous LICS state (where both frequencies were equal at timestep 0). We see that the LICS does not vanish immediately in the new environment (persisting briefly in the yellow highlighted area), though it improves steadily. At the end of these rehabilitation simulation runs, agent cyan's perceived blushing frequencies are readily distinguishable once more: the LICS has been cured.

Taking into account also the findings from Section 3.1.3, that the ability to learn the correct blushing likelihood ratio only slowly decreases with the growing amount of misinformation in the system from 50% onward (left half of Fig. 7), we can conclude that the recovery effect can be expected to be quite stable in even less‐honest environments (although becoming increasingly unreliable and risky). However, the absence of a 0%‐honest agent (and instead the presence of two semi‐honest agents) is crucial, as we saw earlier that the pure deceptiveness of an agent substantially increases the surrounding agents' risk of falling for an LICS (Fig. 5). Further, the removal of a dominant communication strategy might also help for the recovery, but is probably not the main driver.

**Fig. 10:**
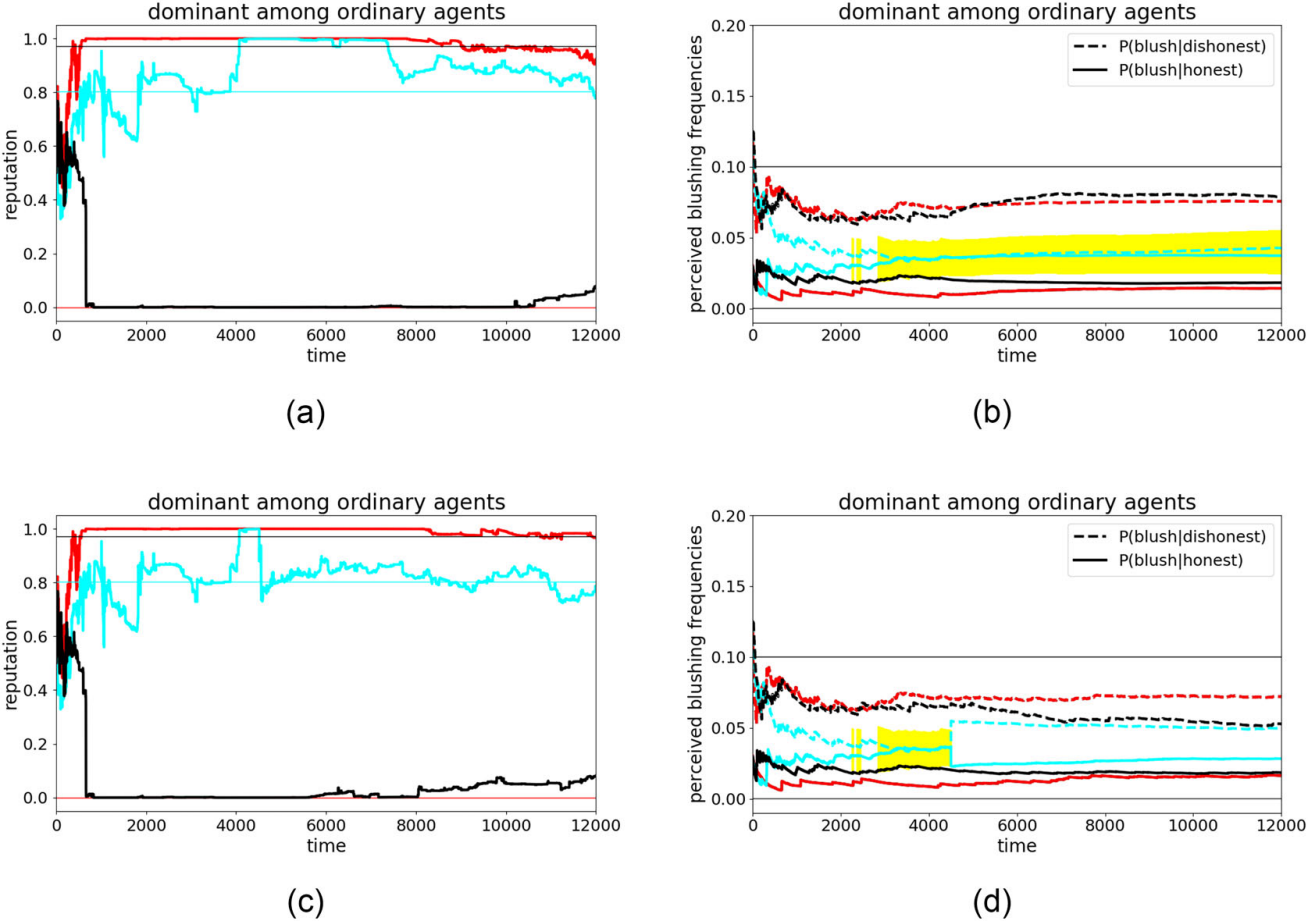
The simulation from Fig. 4c,d that resulted in an LICS is continued here for additional 7500 timesteps. These plots show the average evolution across 20 continuation runs for (a, c, or left) reputation dynamics and (b, d, or right) agents' estimated blushing frequencies. (a, b, or top) show the baseline simulation, where the continuation starts at exactly the state the original simulation ended; (c, d, or bottom) show the subsequent development of a rehabilitated agent cyan. Cyan was removed from this environment for recovery (i.e., put into a new, healthy environment, cf. Fig. 9), and then reintroduced into this toxic environment. The continuation thus starts with a different internal state of agent cyan, namely, its state after the rehabilitation simulation. This explains the sudden change of its estimated blushing frequencies at time 4500.

At this point, one might wonder whether an agent suffering from LICS might also be rehabilitated without changing environments. We, therefore, compare two scenarios: The baseline (unrehabilitated) scenario is a direct continuation of the simulation in Fig. 4c,d beyond timestep 4500, without any intervention, except that we rerun the continuation 20 times to observe its typical further evolution where the previous simulation ended. The results of this baseline continuation can be seen in Fig. 10a,b.

Fig. 10c,d also shows a continuation of agent cyan in its original toxic environment, except that it now has a different internal state after timestep 4500. Here, its continued evolution after timestep 4500 is not a direct continuation from the previous run, but rather represents its reinsertion back into this environment *after* its rehabilitation during the simulation represented by Fig. 9. Agent cyan's internal parameters instantly jump at timestep 4500 in this visualization as we aligned the continuation timesteps with those of the baseline (i.e., left out the interposed rehabilitation run).

Comparing both scenarios yields two main observations. First, contrasting perceived blushing frequencies, we see that rehabilitation simulations indeed led to a stable distinctiveness in agent cyan's perceived frequencies once reinserted into its original toxic environment (10d), whereas in the baseline simulations, agent cyan remains stuck in an LICS for the entire continuation period (10b). Although agent cyan's blushing frequencies are now more stable than at timesteps <4500, there is still a tendency for the two frequencies to converge, meaning the lies of agent red are undetectable by cyan, and can still influence its worldview.

Second, despite this difference in perceived blushing frequencies, the reputation dynamics are very similar with or without rehabilitation (10a,c). Although agent cyan starts to doubt the high reputation of agent red a little earlier, and also improves its low estimation of agent black, these differences are not substantial enough to think that it now has a well‐calibrated estimate of others' honesties.

Overall, then, although an LICS can indeed be cured if the affected individual comes into a new, healthy environment and is no longer under toxic influence, renewed contact with the latter is pernicious, and the affected individual falls back into old patterns in terms of perceived honesty, if not perceived cues of credibility.

## 4 Discussion

In this simulation study, we investigated the dual‐learning problem whereby individuals in social networks must evaluate information given by some sources and, at the same time, establish those sources' credibility. We explored the risk of individuals in such a system falling into delusion‐like states, developing emergent cognitive biases as the result of social influence. In particular, we focused on the emergence of an LICS bias.

With only a few modifications to our previous agent‐based model, the RGS, we examined such scenarios and their underlying dynamics. We showed that agents were able to successfully deal with the dual‐learning problem, typically performing as well at forming an accurate picture of the world (here, estimating others' honesty) as agents who a priori knew the evidential value of credibility cues (i.e., that blushing signs accompanied a certain proportion of lies).

Intriguingly, agents succeeded even in environments where the majority of all communication consisted of lies. This shifts the focus away from misinformation itself (including questions about its prevalence or how to correct it, which occupy so much of the literature). Instead, our results highlight how the problem lies in the interaction between social contexts and individual information processing, given uncertainty both about the world and about cues to credibility. Since the agents in this simulation are rational (albeit imperfect) reasoners, it is especially worth noting that even under such idealized conditions, agents sometimes fail to learn the evidential value of blushing signs.

In our model, delusion‐like states emerged as a persistent and prevalent issue only when agents had to learn cues to credibility in the presence of fully deceptive, malicious agents partly also using specialized communication strategies analogous to flattery, boasting, or bad‐mouthing others. While the specific effects of each strategy varied, we observed that manipulative agents, exhibiting Machiavellian‐like tactics, and dominant agents, displaying traits akin to narcissism, played a key role in shaping group dynamics. Some deceptive strategies increased the likelihood of other agents developing a persistent LICS bias directly, while others did so indirectly by altering trust relationships within the network. Given that deception, flattery, and reputation manipulation are commonly observed in political and competitive social environments, our findings may offer insights into how strategic misinformation can shape collective belief formation.

Further, we demonstrated that agents in a delusion‐like state were reliably able to recover when placed in a new, healthy environment. This environment could still contain a substantial amount of dishonesty (and thus does not call for completely unrealistic settings), as long as it lacked the full deceptiveness of a single malicious agent. The recovery—better‐calibrated estimates of the evidential value of credibility signs—was strong enough to persist even when agents were reintroduced into their old social milieu. However, as estimates of others' honesty involve the interaction of prior knowledge and credibility cues, rehabilitation of the latter was not enough for agents to update their previously mistaken estimates of the former.

While it is important to keep in mind that all results from this simulation resulted from a highly idealized and simplified social system, this demonstrates the value of the presented insights at the same time: In a very controlled setup, we were able to identify a set of features that led individuals to fail in learning reliable signals of credibility, causing them to rationally disregard valuable information.

### 4.1 Limitations and future research

Our simulation represents a highly simplified social system: There are only three agents and they communicate solely about each others' honesty. Also, only one of them uses a malicious strategy at a time, which is perhaps unlikely if people realistically use mixed strategies. Further, real‐world interactions involve larger and more complex networks where individuals discuss multiple topics across different contexts and in different social environments. More realistic malicious strategies might be more versatile and flexible, according to current social contexts. An ideal next step to validate these simplifications would be to apply the model to a real‐world case study. However, we are not aware of any dataset that tracks the development of credibility judgments over time across diverse social environments, and experimentally placing individuals in new social contexts would be difficult. A more feasible approach would be to conduct a behavioral experiment with a setup similar to our simulation, where real participants interact with preprogrammed agents to test whether human responses to manipulative strategies align with our model's predictions. Given these limitations, we understand our work as proof‐of‐concept, or a “how‐possibly” account in the sense of Cailin O'Connor (O'Connor, 2019), illustrating a possible mechanism by which rational agents learn to ignore the evidential value of credibility signs due to social influence. Furthermore, though this simple setup represents a limitation for the applicability to real‐life situations, one advantage is that it allows us to examine certain behavioral patterns in isolation so that we can more clearly trace their mechanisms of action, which is not possible in natural context experiments due to their complexity. Still, in future research, extensions could incorporate larger agent networks, a broader range of discussion topics, or various (deceptive) strategies influencing each other in order to make the model more realistic and more applicable to real‐world scenarios. In particular, the limitation to 3‐agent systems is a major design decision in the current work. While we assume that LICS would also emerge in larger systems, the specific dynamics may not scale directly.

Several factors would likely influence the outcomes in more complex networks: (1) design choices on the network structure could impact how LICS spreads and thus have to be taken carefully; (2) the special strategies (more their technical implementations rather than their general strategy) presented here may not be optimized for larger groups yet and adaptions would have to be made and justified; (3) the overall effect might be weaker as agents have access to a greater number of nonmalicious contacts to recalibrate their reasoning; and (4) LICS could potentially propagate among ordinary agents rather than being confined to direct interactions with deceptive agents, which has not been addressed here at all. These considerations suggest that studying LICS in larger networks requires a separate investigation to assess how our findings generalize to more complex social environments. Nevertheless, these considerations do not diminish the central argument presented here: that social influence can cause agents to disregard reliable evidence of honesty.

Beyond these methodological considerations, our framework offers a mechanistic perspective on how belief in misinformation—including susceptibility to conspiracy‐like thinking—can be shaped by social influences rather than merely by pre‐existing individual cognitive biases. This suggests that instead of focusing solely on the prevalence of misinformation or cognitive predispositions, future research should examine how malicious actors, such as trolls or bots (Evanega, Lynas, Adams, Smolenyak, & Insights, 2020; Geissler, Bär, Pröllochs, & Feuerriegel, 2023), exploit nonepistemic deception strategies to distort credibility judgments.

Current research on misinformation tends to emphasize identifying misleading content and providing corrective interventions (O'Mahony, Luukkanen, Vehmas, & Kaivo‐oja, 2023; van der Linden et al., 2021), often targeting individuals rather than considering the broader social environments in which they learn credibility cues. However, as our rehabilitation simulations demonstrated, merely recalibrating credibility cues was insufficient to correct prior misjudgments about deceptive individuals. This suggests that a sole focus on misinformation cues, without addressing the social structures that reinforce mistaken beliefs or even the mistaken beliefs themselves, may be ineffective in the long term. Given the difficulty of altering one's social environment—especially in an era of constant digital connectivity—systemic problems require systemic solutions.

Further research could explore strategies to prevent individuals from reverting to old biases when repeatedly exposed to manipulative social influences. Since studying such effects empirically is challenging, validated agent‐based models can help fill this gap. For instance, large‐scale fake news detection is feasible due to the visibility of prominent disinformation sources, but identifying deception within smaller, private networks is far more complex. Our findings suggest that even in minimal social systems, individuals can become systematically misled, implying that small‐scale personal networks may be just as consequential as large‐scale media ecosystems in shaping belief formation.

### 4.2 Conclusions

Agent‐based models such as the RGS, by taking into account both the individual psychology of agents and their effects on group dynamics, can be a valuable tool for addressing societal challenges around misinformation, including disbelief in information with robust and objective empirical support such as scientific consensus. In particular, we show how malicious communication substantially increases the emergence of LICS biases, and how this is not simply a matter of widespread misinformation. These findings highlight the importance of healthy social networks which need not be very honest, but should rather strive to decouple signals of credibility from nonepistemic aspects of social influence, especially when individuals are simultaneously uncertain about the world and about others' trustworthiness Supporting Information.

## Funding

Sonja Utz is a member of the Machine Learning Cluster of Excellence, funded by the Deutsche Forschungsgemeinschaft (DFG, German Research Foundation) under Germany's Excellence Strategy

EXC number 2064/1‐Project number 390727645.

Torsten Enßlin is a member of the ORIGINS Cluster of Excellence, funded by the Deutsche Forschungsgemeinschaft (DFG, German Research Foundation) under Germany's Excellence Strategy

EXC number 2094‐Project number 390783311.

## Supporting information

Fig. S1: Histograms of likelihood ratios R(o = b) for different strategies.
Fig. S2: A hypothesis on the typical process that lets deceptive agents achieve a high reputation and also allows them to maintain it permanently.
Fig. S3: Correlations between various parameters that demonstrate the working mechanism of deception strategies.
Fig. S4: Statistics of the emerged LICSs for different strategies played by agent red.

## Notes

1 Nonetheless, misinformation is not just a modern problem (Jin et al., 2024), and it is important not to overstate the prevalence of misinformation online (Altay, Berriche, & Acerbi, 2023).

2 We have deliberately designed the model so that honesty is the only topic of conversation and no other external topic is introduced to keep it as simple as possible. However, one can argue that the system's dynamics should not change much even if we introduced such an external topic: From the model's perspective, one can understand such an extra topic as an additional agent that the others are talking about, but that does not contribute to the conversations itself. One can think of letting this agent give some hints (e.g., via blushing in the model's current setup) throughout the simulation to introduce some data about this topic, or giving the others prior knowledge that they want to convince the others of. In any case, this scenario describes the limit of an agent in the current model setup that does not influence the others' opinions actively. We thus argue that the effects we observe would be very similar whether or not such an external topic is introduced.

3 In the Theory of Mind (ToM), the agents keep track of others' opinions. They do so by evaluating the others' statements throughout the simulation not only in order to update their own knowledge, but also to learn what others currently think, also considering that these statements could only be believed to a certain degree. That is, whenever they hear an (at least to a certain degree) honest message, this will be integrated into their ToM as the speaker's current belief on a topic, while previous beliefs fade out. For example: when Alice (a) talked to Bob (b) about Carol (c), Bob updates his estimate on what Alice believes about Carol, $I_{bac}$, according to $I_{bac}^{\prime }=y_{J}J+\left(1-y_{J}\right)I_{bac}$, where J is the just communicated message and $y_{J}$ is the credibility that Bob assigns the message. How $y_{J}$ is calculated is explained in more detail in A.2. Thus, in case, Bob is absolutely sure that the message was honest ($y_{J}=1$), he can update his ToM about Alice fully as she apparently communicated her true belief. In case of a sure lie ($y_{J}=0$), Bob can not learn anything from the message as it had nothing to do with Alice's true belief and his ToM is not updated. All unclear cases in between are interpolated linearly (Enßlin et al., 2022). Note: This estimation of others' beliefs usually works better when estimating honest agents' beliefs than dishonest ones' as more honest agents (assuming that they are also perceived as honest) reveal more of their true beliefs than dishonest agents do.

4 The amount α by which either of the parameters is increased is governed by two considerations: it should be large enough to have an effect on the receiver's opinion, and at the same time, be small enough to go undetected as a lie—still appearing plausible in the eyes of the receiver. The optimal value for this trade‐off depends on the current “social atmosphere” the agents find themselves in, or how surprising the messages are in general at the moment. For instance, if messages are typically very chaotic and spreading a lot, a lie can be larger without being noticed. If, however, the agents pretty much agree and opinions are settled, lies must be smaller. Each agent i measures this atmosphere by a parameter $\kappa_{i}$, which is the median of the surprise values (given by the Kullback–Leibler Divergence ($D_{\mathrm{KL}}$) (Kullback & Leibler, 1951)) of the last 10 received messages. Assuming that the normalized surprise of an honest message $S_{J}$ follows a simple exponential distribution $P\left(S_{J}|h\right)=\exp\left(-S_{J}\right)$, which the agents want to imitate with their lies, they choose the lie size α such that the receiver's surprise meets a typical surprise value $S_{J}$ drawn from this distribution: $D_{\mathrm{KL}}\left(\mathrm{Lie}\left(\alpha \right)|I_{abc}\right)=\kappa_{a}f_{\mathrm{caution}}S_{J}$. Here, the lying agent a has to replace the receiver's perception of the atmosphere $\kappa_{b}$ with its own, which is very similar in most cases, though, and we introduced the factor $f_{\mathrm{caution}}=0.3$ as it turned out that the lies would be too risky otherwise and work better if they are smaller (Enßlin et al., 2022). In short, the size of lies depends on the current social atmosphere and is stochastically drawn to imitate the typical surprise of an honest statement.

5 As the Kullback–Leibler Divergence (KL) measures the amount of information needed to update from one to another probability distribution (Kullback & Leibler, 1951), the agents minimize the KL, that is, the amount of lost information when updating from the correct posterior to the simplified version.

6 This holds for ordinary agents. Special agents use a fourth criterion, called expectation matching: they compare the message with their current estimation of the speaker's true belief and what the speaker typically lies to them. Depending on how close the message is to either of the two reference points, the message's credibility continually in‐/decreases. The agents keep track of what the speaker typically says when lying to them, using same update algorithm as for the ToM (see footnote 3) but for dishonest statements. For mathematical details, see footnote 14 in the Appendix or Enßlin et al. (2022). However, this criterion turned out to have only little impact on the simulation results and is thus not of further importance here. We still retain it in order to have comparable results to previous work (Enßlin et al., 2022; Kainz, Bœhm, Utz, & Enßlin, 2022; Enßlin, Kainz, & Bœhm, 2023).

7 Even if agents are certain about whether they themselves have lied or told the truth, their self‐knowledge is just one source of information about the meaning of blushes. False assessments of the other agents can thus prevent them from learning perfectly from their self‐observations, wherein blushes only occur with lies.

8 The specific values do not matter and are drawn randomly, though for our core example, we aimed for a setup with two mostly honest agents and one mostly dishonest agent in order to investigate the effect of one malicious agent on a typically cooperative (in our case, “honest”) society. Following research on the evolution of stable communication (Scott‐Phillips, 2011; Schneier, 2012), the assumption of a generally honest signaling system seems reasonable. The effect of different honesty values is discussed below in Section 3.1.3 and even more in the Supplementary Material in the section “Special Strategies in Less Honest Environments.”

9 We define the informedness of an agent i as the alignment of all agents' true honesties $x_{k}$ with agent i’s estimation of them, $x_{ik}$. Thus, agent i’s informedness is $\sum_{k}\left(x_{ik}-0.5\right)\cdot \left(x_{k}-0.5\right)$, where the sum covers over all agents k in the simulation.

10 Agents do not know their own intrinsic honesty but have to learn it from their experiences throughout the simulation. In general, this works well as they can observe themselves making honest/dishonest statements and can count, but they might as well be influenced by others' statements about themselves that potentially lead to wrong self‐images.

11 Indeed, any remedy that required a completely honest society would be useless. Moreover, the presence of lies is essential to learn to classify blushing signals correctly, because otherwise, there would be no evidence at all to allow any interpretation of blushing signals.

12 We do not show the summary of reputation dynamics across multiple simulation runs for this rehabilitation scenario. As the honesty of agents yellow and green is randomly distributed over simulations, they would both reach a value close to 0.5 on average, and so would their reputation. The corresponding graph would, therefore, not be instructive.

## Appendix A Learning the significance of credibility signs

While the frequency with which the agents accidentally reveal their lies by blushing is fixed at $f_{b|\neg h}=0.1$, the agents do not know this a priori, but have to learn the frequency. Also, the agents could, in principle, think that someone blushes when making an honest statement (although this is not possible, i.e., $f_{b|h}=0$), which leads to a second frequency the agents have to infer from observations.

### A.1. Setup

Each agent stores its own estimate of the perceived blushing frequencies in honest and dishonest statements, $f_{b|h}$ and $f_{b|\neg h}$. Implicitly, we assume hereby that each agent has an individual perception of the expressiveness of blushing signs, which may deviate from agent to agent. Furthermore, we assume that the agents do not distinguish between the blushing signs they receive from the individual other agents, but interpret all blushings they receive from the different agents as a similarly strong dishonesty signal.

Since the experience of agents can be viewed as counting honest and dishonest statements that appeared with or without blushing, that is, counts of binary events, the natural choice for a probability density function summarizing the obtained information on the blushing frequencies is a beta distribution. Thus, we define:

(A.1)
$$\begin{array}{c} \begin{array}{cc} P(f_{b|h}|\mu_{b|h},\lambda_{b|h}) & =\text{Beta}\left(f_{b|h}|\mu_{b|h}+1,\lambda_{b|h}+1\right)=\frac{f_{b|h}^{\mu_{b|h}}{\left(1-f_{b|h}\right)}^{\lambda_{b|h}}}{B(\mu_{b|h}+1,\lambda_{b|h}+1)}\\ P(f_{b|\neg h}|\mu_{b|\neg h},\lambda_{b|\neg h}) & =\text{Beta}\left(f_{b|\neg h}|\mu_{b|\neg h}+1,\lambda_{b|\neg h}+1\right)=\frac{f_{b|\neg h}^{\mu_{b|\neg h}}{\left(1-f_{b|\neg h}\right)}^{\lambda_{b|\neg h}}}{B(\mu_{b|\neg h}+1,\lambda_{b|\neg h}+1)}, \end{array} \end{array}$$

with B being the beta function. The means of the distributions ${\bar{f}}_{b|h},{\bar{f}}_{b|\neg h}\in \left[0,1\right]$ are the agents’ momentary best guesses of the blushing frequencies in honest and dishonest statements, respectively.

At the beginning of a game, the agents are equipped with a prior belief on both frequencies that represent their true values but also contain some uncertainty. Since the agents are able to self‐calibrate the assumed blushing frequencies by observing themselves the prior can be weak, allowing for an unbiased, dynamical evolution of the parameters.^13^ Therefore, we use slightly informative initial priors for both frequencies, which can be understood as 30 correct observations the agents have made prior to the simulation for the possible cases each:

(A.2)
$$\begin{array}{c} \begin{array}{cc} P_{\text{prior}}\left(f_{b|h}\right) & =P(f_{b|h}|\mu_{b|h}=0,\lambda_{b|h}=30)\\ P_{\text{prior}}\left(f_{b|\neg h}\right) & =P(f_{b|\neg h}|\mu_{b|\neg h}=3,\lambda_{b|\neg h}=27). \end{array} \end{array}$$

### A.2. Credibility estimation

The blushing frequencies, or more precisely speaking the agents' current estimations thereof, then enter the learning mechanism by influencing the agents' credibility assessment of a received message. In order to draw correct conclusions from a conversation and to gain as much (correct) information as possible, the agents have to estimate to which degree they can believe the message and to which degree it should be regarded as an attempted deception of the speaker. The receiving agent thus calculates the probability $y_{J}$ of the message J being honest h given certain data d=(J,o), where o∈{b,¬b} is the observation whether or not the speaker blushed. Following Bayes Theorem, we derive:

(A.3)
$$\begin{array}{c} \begin{array}{cc} y_{J} & =\frac{P(d|h)P(h)}{P(d)}={\left[1+R\left(d\right)(P{\left(h\right)}^{-1}-1)\right]}^{-1}\;\;\;\;\text{with}\;\;\;\;R\left(d\right)=\frac{P(d|\neg h)}{P(d|h)}. \end{array} \end{array}$$

Besides the general probability of the speaker being honest P(h) as seen by the receiver, that is, the latter's current opinion on the speaker's honesty, the credibility is defined by the likelihood ratio R. Generally, the receivers use three (or four in the case of special agents) independent criteria for its estimation: blushing, confessions, and the surprise of the message (and expectation matching). Thus, we can write:

(A.4)
$$\begin{array}{c} R\left(d\right)=\prod_{j}R_{j}\left(f_{j}\left(d\right)\right)=\prod_{j}\frac{P(f_{j}\left(d\right)|\neg h)}{P(f_{j}\left(d\right)|h)} \end{array}$$

for each of the features $f_{j}$. For the sake of this work, however, we only detail out the mathematical description of the blushing feature, as this is the one leading to LICS.^14^ In theory, if the agents knew the exact blushing frequencies $f_{b|h}=0$ and $f_{b|\neg h}=0.1$, the likelihood ratio for blushing would be given by:

(A.5)
$$\begin{array}{c} R_{\text{b, theory}}\left(o\right)=\left\{\begin{array}{cc} \frac{P(b|\neg h)}{P(b|h)}=\frac{f_{b|\neg h}}{f_{b|h}}=\frac{0.1}{0}=\infty , & o=b\\ \frac{P(\neg b|\neg h)}{P(\neg b|h)}=\frac{1-f_{b|\neg h}}{1-f_{b|h}}=\frac{0.9}{1}=0.9, & o=\neg b \end{array}\right.. \end{array}$$

This means that whenever the receiving agent observes the speaker blushing, $R_{b}\left(o=b\right)=\infty$ and thus $y_{J}=0$, that is, the receiver can be sure that the message was a lie. However, when the speaker did not blush, $R_{b}\left(o=\neg b\right)=0.9$, which has a positive influence on $y_{c}$ as the absence of a blush is a weak hint toward an honest statement.

In reality, however, the evidential value of such an evidence is not a priori clear and has to be learned. This is what we simulate here by letting the agents learn the two blushing frequencies $f_{b|h}$ and $f_{b|\neg h}$, which will result in an individual interpretation of blushing signs based on previous experiences. The likelihood ratio R that defines how an observed blushing sign is evaluated thus takes the more general form:

(A.6)
$$\begin{array}{c} \begin{array}{c} R_{\text{b, reality}}\left(o\right)=\left\{\begin{array}{cc} \frac{P(b|\neg h)}{P(b|h)}=\frac{{\bar{f}}_{b|\neg h}}{{\bar{f}}_{b|h}}, & o=b\\ \frac{P(\neg b|\neg h)}{P(\neg b|h)}=\frac{1-{\bar{f}}_{b|\neg h}}{1-{\bar{f}}_{b|h}}, & o=\neg b \end{array}\right., \end{array} \end{array}$$

where the overbar indicates that these are now the agents' current estimations of blushing frequencies at any point in time of the simulation and not their actual, correct values.

### A.3. Updating the significance of blushing

The remaining question is though, how the agents learn the blushing frequencies throughout the simulation: After estimating the credibility of a received message $y_{J}$ (and updating their knowledge about the speaker and topic accordingly), the agents also update their knowledge about the blushing frequency parameters. In general, they do so by comparing the observation whether or not the speaker blushed and their estimation of whether or not the speaker lied. In case the receiver is absolutely sure that the message was honest, that is, $y_{J}=1$, it can either account for one more blushing (no blushing) in honest statements if the receiver blushed (did not blush). The updated probability distribution about the parameter $f_{b|h}$ is thus given by:

(A.7)
$$\begin{array}{cc} P^{\prime }\left(f_{b|h}|o\right) & =\left\{\begin{array}{cc} P(f_{b|h}|\mu_{b|h}+1,\lambda_{b|h}), & o=b\\ P(f_{b|h}|\mu_{b|h},\lambda_{b|h}+1), & o=\neg b \end{array}\right., \end{array}$$

while there can be no update on $f_{b|\neg h}$ as no new information about it is available. In the other case, where the receiver is sure that the speaker lied, that is, $y_{J}=0$, it should update

(A.8)
$$\begin{array}{cc} P^{\prime }\left(f_{b|\neg h}|o\right) & =\left\{\begin{array}{cc} P(f_{b|\neg h}|\mu_{b|\neg h}+1,\lambda_{b|\neg h}), & o=b\\ P(f_{b|\neg h}|\mu_{b|\neg h},\lambda_{b|\neg h}+1), & o=\neg b \end{array}\right., \end{array}$$

but there is no information on $f_{b|h}$. Since, however, in most cases, the status of a message, whether it has been honest or not, can not be taken as granted, that is, $y_{J}$ is somewhere between 0 and 1, the resulting update of the blushing frequencies will be a superposition of both possibilities

(A.9)
$$\begin{array}{c} \begin{array}{cc} P^{\prime }\left(f_{b|h}|o\right) & =y_{J}\left\{\begin{array}{cc} P(f_{b|h}|\mu_{b|h}+1,\lambda_{b|h}), & o=b\\ P(f_{b|h}|\mu_{b|h},\lambda_{b|h}+1), & o=\neg b \end{array}\right\}+\left(1-y_{J}\right)P\left(f_{b|h}|\mu_{b|h},\lambda_{b|h}\right)\\ P^{\prime }\left(f_{b|\neg h}|o\right) & =y_{J}P\left(f_{b|\neg h}|\mu_{b|\neg h},\lambda_{b|\neg h}\right)+\left(1-y_{J}\right)\left\{\begin{array}{cc} P(f_{b|\neg h}|\mu_{b|\neg h}+1,\lambda_{b|\neg h}), & o=b\\ P(f_{b|\neg h}|\mu_{b|\neg h},\lambda_{b|\neg h}+1), & o=\neg b \end{array}\right\}. \end{array} \end{array}$$

Similarly to the update about others honesties (as described in Section 2), these superpositions have to be compressed into a single beta distribution that the agents can remember. This is again done via minimizing the KL Divergence between the full, superpositional posterior and a single beta distribution.

Note that the agents also observe themselves talking and blushing, which gives them at least some certain data for this inference that simplifies the agents' learning.
