# Supplementary material for: Learned Insignificance of Credibility Signs
Source: Cogn Sci. 2025 Aug 14;49(8):e70102. doi: 10.1111/cogs.70102 (PMC12351531; doi:10.1111/cogs.70102)
Supplement: Supplementary file 1 — Fig. S1: Histograms of likelihood ratios R(o = b) for different strategies. Fig. S2: A hypothesis on the typical process that lets deceptive agents achieve a high reputation and also allows them to maintain it permanently. Fig. S3: Correlations between various parameters that demonstrate the working mechanism of deception strategies. Fig. S4: Statistics of the emerged LICSs for different strategies played by agent red. [file COGS-49-e70102-s001.pdf]

Learned Insignificance of Credibility Signs

June 28, 2025

1 Learning the Significance of Credibility Signs

The likelihood ratio $\mathcal{R}$ that defines how an observed blushing sign is evaluated is given by

$$\mathcal{R}_{\mathrm{b}}(o) = \begin{cases} \frac{P(\mathrm{b}|\neg\mathrm{h})}{P(\mathrm{b}|\mathrm{h})} = \frac{\overline{f}_{\mathrm{b}|\neg\mathrm{h}}}{\overline{f}_{\mathrm{b}|\mathrm{h}}}, & o = \mathrm{b} \\ \frac{P(\neg\mathrm{b}|\neg\mathrm{h})}{P(\neg\mathrm{b}|\mathrm{h})} = \frac{1-\overline{f}_{\mathrm{b}|\neg\mathrm{h}}}{1-\overline{f}_{\mathrm{b}|\mathrm{h}}}, & o = \neg\mathrm{b} \end{cases}, \tag{1}$$

where $\overline{f}_{\mathrm{b}|\mathrm{h}}$ and $\overline{f}_{\mathrm{b}|\neg\mathrm{h}}$ are the agents' current estimations of the blushing frequency in honest and dishonest statements, respectively. With the two blushing frequencies ranging from 0 to 1 the likelihood ratio can now in principle take any positive value and Figure 1 shows the statistics of actually emerged, i.e. learned likelihood ratios $\mathcal{R}(o = \mathrm{b})$. We see that especially when agent red played the deceptive, manipulative or dominant strategy the ordinary agents' blushing likelihood ratios are significantly shifted to smaller values, indicating a higher risk for a LICS. This is also consistent with the higher number of LICSs for those strategies, shown in the main work. Additionally, we can observe effects of the high-risk-/high-gain dynamics that a dominant agent red produces, which was discussed in the main text as well. Compared to the deceptive agent, which shows a smooth distribution of likelihood ratios (in particular at the left edge) that is basically just a shifted version of the ordinary case, there is a clear second shoulder visible for the simulations with a dominant agent. This results from the fact that the dominant strategy either is fully successful in some runs, i.e. leads to top reputations for agent red and thus maximal delusion of the ordinary agents or is not effective at all in other runs.

2 Achieving High Reputations

The overall mechanism that makes deceptive agents achieve and maintain a high reputation is schematically illustrated in Figure 2, which is an extension of what has already been shown in the main work. Here we want to additionally discuss how deceptive agents achieve high reputations in the first place, i.e. the left, linear part of the diagramm[1]. For the sake of simplicity we do not look at all special strategies individually, but rather at their common characteristics. The unifying property of all special strategies presented here (manipulative, dominant, destructive) is their deceptiveness, i.e. absolute dishonesty, and their sophisticated lie-detection-mechanism. An agent embodying these characteristics is what we call a **clever agent**. As shown in [1], deceptiveness already leads to a high informedness compared to all non-deceptive agents. The reason is that the non-deceptive agents never have a chance to learn the true beliefs of deceptive agents, can therefore only poorly target their (anyhow less frequent) lies at them, and therefore are easier caught lying by the deceptive agents. This way, deceptive agents can better filter out false information, which makes them better informed on the one hand and also improves their first order Theory of Mind (ToM) on the other hand. Additionally to their deceptiveness, the special strategies also come with a specialization component. All of them prefer certain conversation topics, which they frequently talk about and therefore also force their conversation partners to do so. Hence, they rapidly build up some expertise about their prefered topic and/or conversation partner, which ordinary agents cannot as they randomly choose their topics and talk about everyone approximately equally often. Also, being better informed as well as having a better ToM are self- and mutually reinforcing states, as both help to detect lies more accurately and thus to use the available information more effectively. This correlation, between informedness and the quality of the agents' ToM can be seen in the upper panels of Figure 3. Both for ordinary agents and a clever agent among ordinaries we can

[1]Parts thereof were already found in previous studies [1, 2] and do not require the effect of a LICS. Nevertheless we want to discuss the full mechanism here fore completeness.

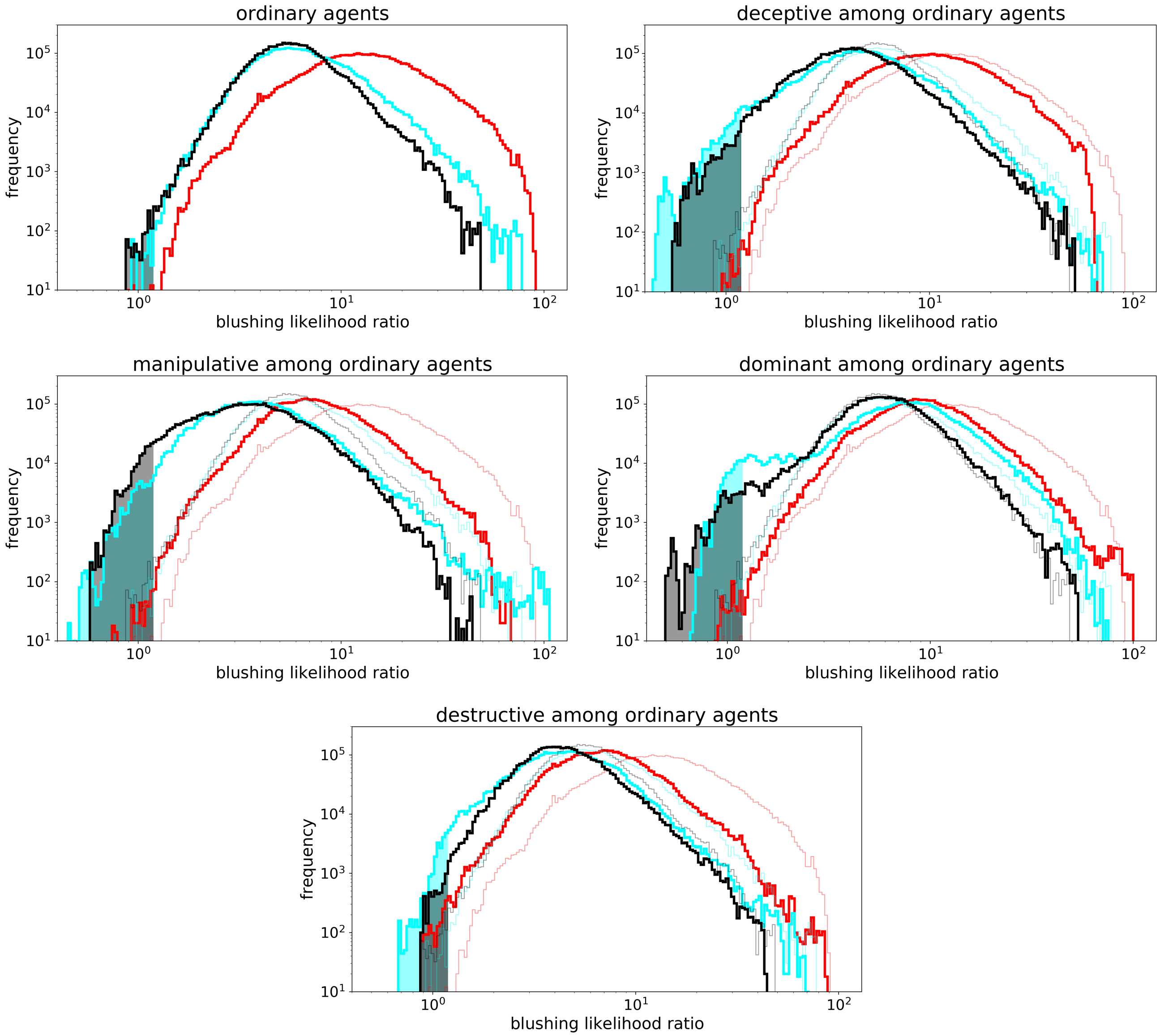

Figure 1: Histrograms of likelihood ratios $R(o = \mathrm{b})$ for different strategies. The thick lines in the different panels show the results for different special strategies played by agent red, which is specified by the titles. Agents cyan and black are always ordinary agents. The likelihood ratios are shown for the individual agents indicated by their colors. The thin lines in each panel are a replication of results with red being an ordinary agent for easier comparison. The filled area indicates all states that we regard as LICS, i.e. states in which $1.1 f_{\mathrm{b}|\mathrm{h}} > 0.9 f_{\mathrm{b}|\neg\mathrm{h}}$ for the agents' current estimation holds.

clearly see that high informedness comes along with a good ToM. When a clever agent is involved, we additonally see a branch where the agents' ToMs are quite accurate, but their informedness is low. This emerges whenever the clever agent managed to achieve a high reputation, i.e., convinces the others of its high honesty (although being fully deceptive). In such a state the worldview of the agents is wrong, i.e., their informedness is low, but at the same time this worldview is stable, i.e. all agents agree on this false belief, which allows for an accurate estimations of others' beliefs, i.e., ToM.

Thanks to being better informed about the others' beliefs, i.e. a better ToM, deceptive agents are correspondingly able to target their lies more precisely. We do not have to show this, as it is imprinted on the simulation dynamics by construction [2]. Moreover, well targeted lies enables the deceptive agents to influence their conversation partner more effectively, meaning that they should profit in terms of reputation. Indeed, we also observe this relation when looking at the middle row of Figure 3, at least for reputations above 0.5. For lower reputations another effect plays

a more important role: whenever a deceptive agent has a low reputation, the others are aware of its low honesty, i.e. do not belief the statements made by the deceptive agent. Thus, its wrong statements do not influence any beliefs including its own, as deception might otherwise come back via an echo-chamber effect. This way the whole system agrees on the low honesty of the deceptive agent and all their ToMs become accurate. In this regime, the causal relation between ToM and reputation is actually inverted.
However, if we are in the high reputation regime of the deceptive agent, we are already in the self-reinforcment circle described in the main work, in which a high reputation gives the statements more weight and ordinary agents belief the deception. We can also observe this correlation appearing in our simulation as the lower panels of Figure 3 show. From here the self-stabilizing circle takes over.
It should be noted, though, that this is an idealized description of the mechanism and cannot be found exactly like this neither in reality nor in the RGS. The true relationship is more complex, less linear and shaped by numerous cross-influences and resulting exceptions. Nevertheless the above description captures the main effects and elemental system dynamics in the RGS on the basis of which all strategy-specific phenomena should be considered.

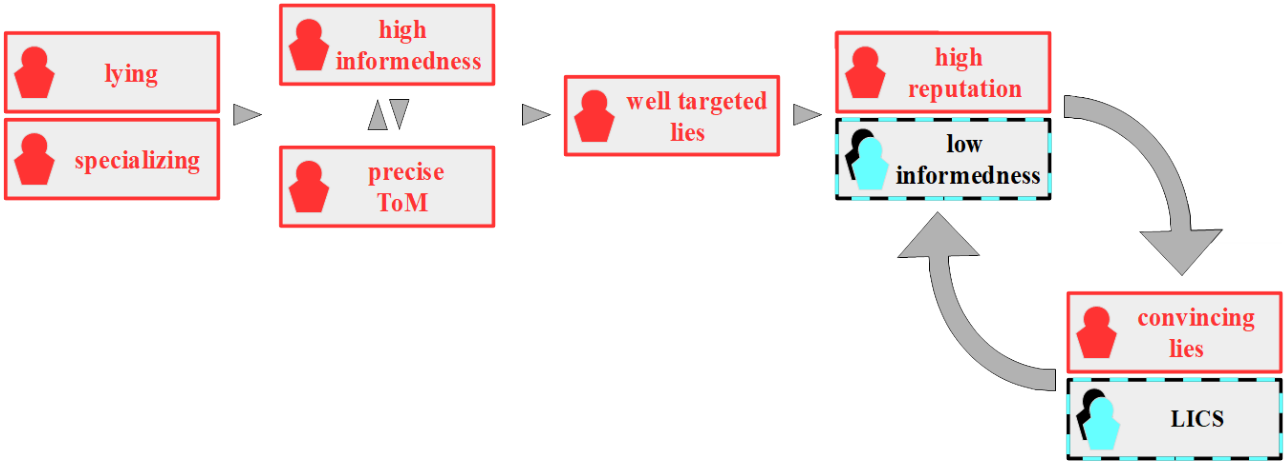

Figure 2: A hypothesis on the typical process that lets deceptive agents achieve a high reputation and also allows them to maintain it permanently. The two properties of being deceptive and specializing in a certain topic (left) makes agents better informed than more honest ordinary agents (cyan, black) [1]. This does not only concern their estimation of others' honesties, but also their first order ToM, i.e., their estimations of others' beliefs. Having a precise, correct picture of others' beliefs, in turn, enables the deceptive agents to construct their lies in a way that makes them sound plausible and believable to the message receivers, who will consequentially believe the lies. Since these lies are in favor of the deceptive agent, their reputation will increase. At the same time the ordinary agents, who believe the lies will become poorly informed as they trust wrong information. Once such a state of a highly reputed deceptive agent and poorly informed ordinary agents is established, a self-amplifying effects keeps it up: The reputed, well informed liar will be believed even more and the ordinary agents piece by piece lose their sense for reality and judgement, i.e., get into a LICS. Consequentially, they will further misinterpret messages and other evidential information such as blushing and stay poorly informed, while the deceptive agent can stabilize its high reputation and the circle can start all over again.

3 Special Strategies in Less Honest Environments

In the main work we only investigate the effect of special strategies in environments of largely honest ordinary agents with honesties of 80% and 97%. While the exact numbers are drawn uniformly from an interval between 0 and 1, we did choose the random seed such that those numbers are rather high. We did so following the assumption that societies usually rely on mainly cooperative individuals (aligned with research on the evolution of stable communication [3, 4]) and we thus argue that mostly honest ordinary agents are a valid choice. Still, we want here to investigate the effect of the same special strategies on less honest, ordinary agents in order to see how the system behaves under these circumstances. Therefore we ran the same set of 1000 simulations with varying random seeds as used in the main work for fixed honesties of agents cyan and black, but instead vary the latters' honesties in each simulation. All three agents' honesties are drawn uniformly from numbers between 0 and 1 and afterwards they are sorted by honesty, such that the color red is assigned to the least honest agent, cyan to the

middle one and black to the most honest agent. This setup is also exactly the same that was already introduced in the main work when discussing the influence of honesty on the emergence of LICS with an average honesty of 50%. Here, we want to additionally study the effect of special strategies played by agent red in this system. For all those strategies, agent red's (randomly drawn) honesty is overwritten and set to 0 as otherwise the strategies' effects would become unnoticeable. Consequentially we end up with a system containing one 0-honesty-agent red, and two ordinary agents with average honesties of 50% and 75%, respectively. The average honesty among ordinary agents thus decreased from 88.5% to 62.5%. In Figure 4 one can see the statistics of emerged LICS for the various strategies. First of all we can note, that LICSs do as well appear in all of these settings, which is not surprising as the overall amount of misinformation in the system grew and learning (including estimating the evidential value of blushing signs) should at least not have become easier. Also, we still see a similar behavior than before when comparing the different strategies among each other: the ordinary agent red still causes least LICS, while the manipulative agent caused most and deceptive, dominant and destructive end up at similar levels. However, there are two main differences when comparing the results with the more honest environment. First, the overall frequency of LICS increased from an average of around 8% to around 17%, and second, this difference is mostly caused by agent red falling for LICS itself. While this may sound counter-intuitive and irritating at a first glance, it can be explained very well with what we already understood from the findings in the main work. Basically, not falling for a LICS requires a stable calibration of the likelihood ratio. For this, the agents need data to learn from, which they have to collect throughout the simulation. Usually (in a pretty honest environment) the red agents have enough and valuable data on both the frequency of blushing in dishonest statements (by their own frequent dishonest statements and self-observations) and the frequency of blushing in honest statements (by observing their mostly honest environment that they are usually able to interpret correctly). Thus, they are able to calibrate the ratio of both frequencies quite reliably and thus do not falls for LICS often. However, when it comes to the new scenario with more dishonest ordinary agents, two important things change: one the one side, there are now less honest statements in the system in general, as the average honesty of agent cyan and black changed from $(80\% + 97\%)/2 = 88.5\%$ to $(50\% + 75\%)/2 = 62.5\%$, which makes it harder to calibrate the blushing frequency in honest statements. One can argue, that at the same time it should become easier to calibrate the blushing frequency in dishonest statements, though - which is definitely the case - but especially for agent red who has no blushing information at all for honest statements from self-observations, the loss of this information is way severe than the gain of information about dishonest statements, which it has enough of anyways. On the other side, the information on blushing signs that is available must also be interpreted correctly. I.e. honest statements must be recognized as honest and dishonest statements must be recognized as dishonest in order to draw the right conclusions from them. Also this becomes more difficult in the new setup as less honest agents are harder to judge, as has as well been observed in previous works on the RGS [1]. Taking both arguments together, the findings above are not surprising anymore, but show very reasonable behavior. Moreover, the findings demonstrate that the here presented special strategies rely on mostly honest ordinary agents around them in order to exploit their tactics fully. In other words, we see here once again that dishonesty can be an effective counter-strategy to dishonesty in the first place, similar to what has been found in [1]. Although this has to be understood with a grain of salt, of course, any deliberately malicious behavior will be adapted to the current environment to which it is applied to and so are our special strategies adapted to a mostly honest surrounding, as this is how one can assume a healthy society to be [3, 4].

References

[1] Viktoria Kainz, Céline Bœhm, Sonja Utz, and Torsten Enßlin. Information and agreement in the reputation game simulation. *Entropy*, 24(12), 2022. ISSN 1099-4300. doi: 10.3390/e24121768. URL `https://www.mdpi.com/1099-4300/24/12/1768`.

[2] Torsten Enßlin, Viktoria Kainz, and Céline Bœhm. A reputation game simulation: Emergent social phenomena from information theory. *Annalen der Physik*, 534(5):2100277, 2022.

[3] Thomas C Scott-Phillips. Evolutionarily stable communication and pragmatics. *Language, games, and evolution*, 6207:117–133, 2011.

[4] Bruce Schneier. *Liars and outliers: enabling the trust that society needs to thrive.* John Wiley & Sons, 2012.

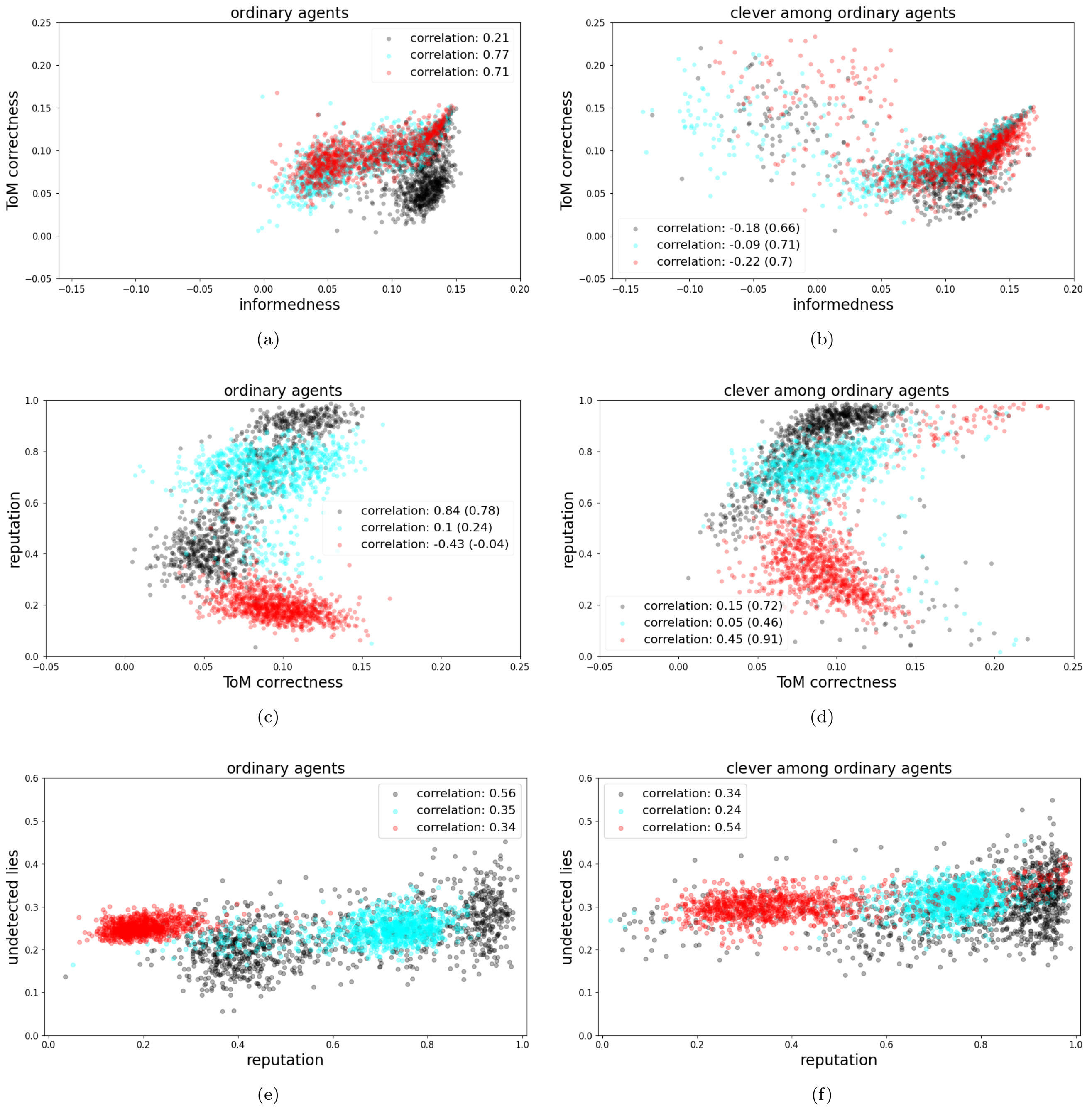

Figure 3: Correlations between various parameters that demonstrate the working mechanism of deception strategies. A dot refers to the time-average of a quantity over the whole simulation and its color corresponds to the agent who holds the quantity. First row (a,b): the correlation between the agents' informednesses, i.e., how well their estimations of others' honesties is aligned with their true intrinsic honesties, and the correctness of their ToM, i.e., how well their estimations of others' opinions is aligned with the others' actual opinions. The correlations in brackets in (b) refer to data points with informedness ≥ 0.07 to distinguish the two regimes visible there. Second row (c,d): the correlation between the agents ToM correctness and their reputation, i.e., its estimated honesty as seen by all others. The correlation values in brackets refer to all points with a reputation ≥ 0.5, again to distinguish between systematically different regimes (see main text for explanations). Third row (e,f): the correlation between the agents' reputation and the percentage of lies they can make undetectedly (defined by the average credibility others assign to that agents' lies). All left panels (a,c,e) show the behaviour of three ordinary agents as a baseline strategy. The right panels (b,d,f) show how the influence of a clever agent (red) changes the system by its deception strategy. This strategy is the basis of all other special strategies shown in this work (manipulative, dominant and destructive) and can be understood as their common thread.

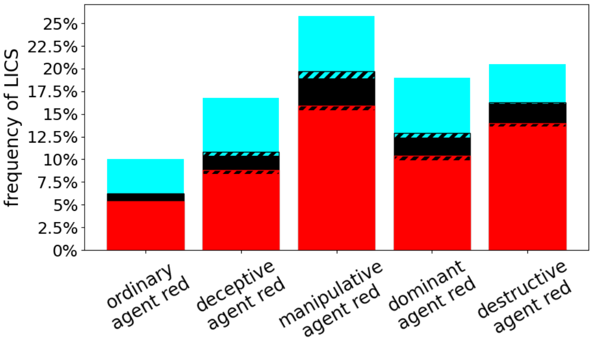

Figure 4: Statistics of the emerged LICSs for different strategies played by agent red. For 1000 simulations with different random seeds the percentage of simulations where at least one of the agents developed a LICS is shown. The distribution of the frequencies among the three agents is shown in their respective colors. The x-axis indicates the strategy played by agent red, while agents cyan and black are always ordinary agents. The honesties of all three agents are drawn uniformly from values between 0 and 1 for each of the 1000 simulations. They are sorted by color, i.e. agent red in every simulation is the least honest agent, agent cyan the one in the middle and agent black is most honest. Note that whenever agent red uses a special strategy that involves an honesty of 0, its previously drawn honesty is overwritten. In some simulations more than one agent develops a LICS, which is indicated by the striped areas. In some rare cases also all three agents develop a LICS in the same simulation, which can not be visualized in this figure: For both the deceptive and manipulative agent red this happened in 0.1% of the cases, for the dominant agent red in 0.3% and for the other strategies not at all.
